# The circadian clock ticks in plant stress responses

**DOI:** 10.1007/s44154-022-00040-7

**Published:** 2022-03-01

**Authors:** Xiaodong Xu, Li Yuan, Qiguang Xie

**Affiliations:** State Key Laboratory of Crop Stress Adaptation and Improvement, School of Life Sciences, Henan University, Kaifeng, 475004 China

**Keywords:** Circadian clock, Temperature stress, Drought, Salinity, Pathogen

## Abstract

The circadian clock, a time-keeping mechanism, drives nearly 24-h self-sustaining rhythms at the physiological, cellular, and molecular levels, keeping them synchronized with the cyclic changes of environmental signals. The plant clock is sensitive to external and internal stress signals that act as timing cues to influence the circadian rhythms through input pathways of the circadian clock system. In order to cope with environmental stresses, many core oscillators are involved in defense while maintaining daily growth in various ways. Recent studies have shown that a hierarchical multi-oscillator network orchestrates the defense through rhythmic accumulation of gene transcripts, alternative splicing of mRNA precursors, modification and turnover of proteins, subcellular localization, stimuli-induced phase separation, and long-distance transport of proteins. This review summarizes the essential role of circadian core oscillators in response to stresses in *Arabidopsis thaliana* and crops, including daily and seasonal abiotic stresses (low or high temperature, drought, high salinity, and nutrition deficiency) and biotic stresses (pathogens and herbivorous insects). By integrating time-keeping mechanisms, circadian rhythms and stress resistance, we provide a temporal perspective for scientists to better understand plant environmental adaptation and breed high-quality crop germplasm for agricultural production.

## Introduction

**Fig. 1:**
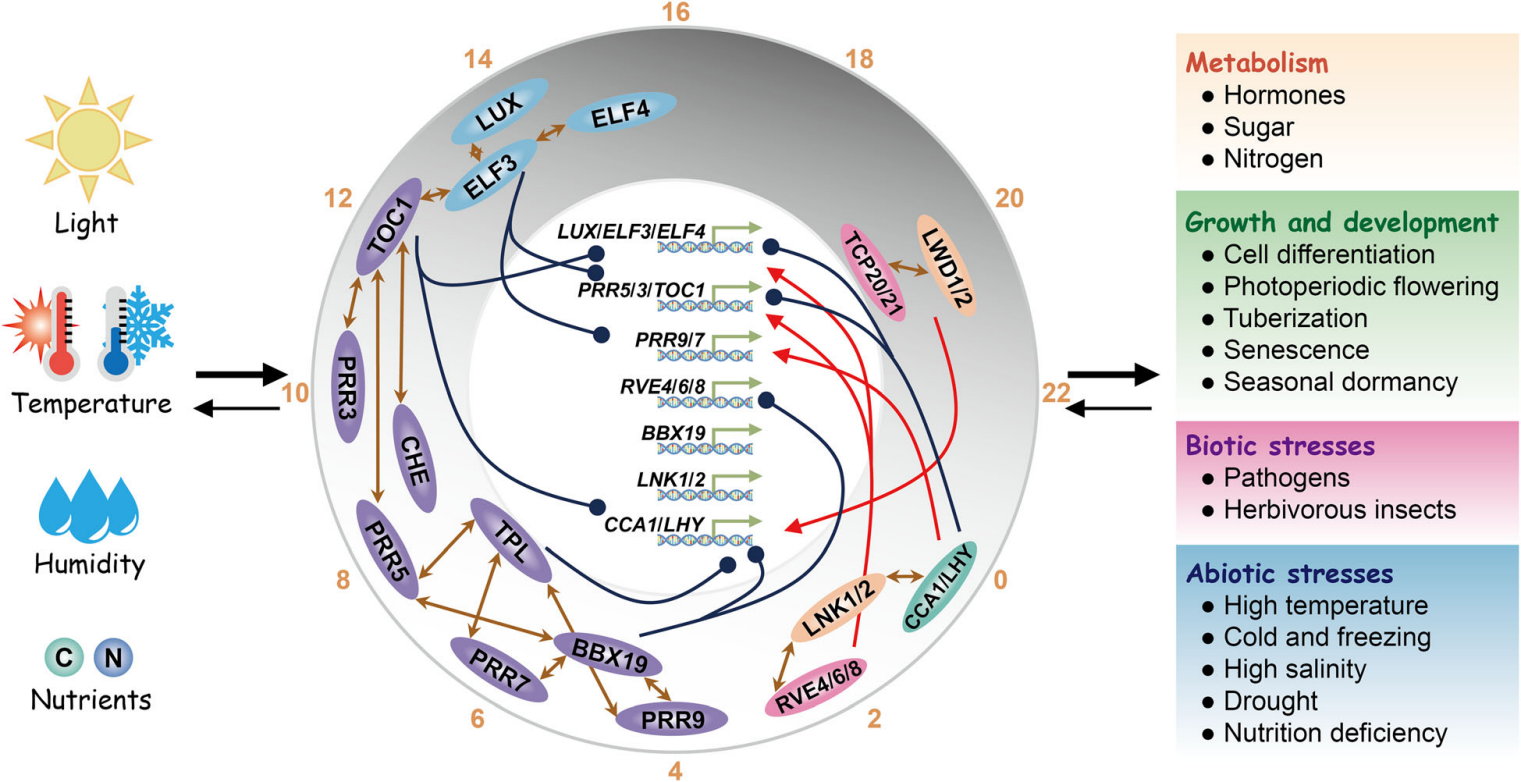
The circadian system in higher plants

The circadian clock allows organisms to anticipate cyclic changes of environmental signals and generate endogenous 24-h rhythms of physiological responses in synchronization with external and internal cues, thereby providing organisms advantages for survival (Nohales and Kay, 2016; Creux and Harmer, 2019; Bonnot et al., 2021). The circadian clock system in plants consists of time-giving input pathways mediated by environmental signals, sequentially expressed core oscillators, and numerous output pathways. The timing givers in the environment are also called “*Zeitgebers*”, which cue time of day or season (Xu et al., 2022b). The expression of core oscillators is regulated by input signal components, and then directly or indirectly act on the rhythmic transcript accumulation of their target genes, thereby generating daily cellular, metabolic, and physiological rhythms or seasonal rhythms (Greenham and McClung, 2015; McClung, 2019; Webb et al., 2019; Sanchez et al., 2020) (Fig. 1). In this review, we will summarize the responses of the circadian clock to the resetting cues, which inevitably involve the extreme temperature and light intensity in day and night, water and nutrition deficiencies, as well as dormancy and freezing tolerance during the transition of seasons. Core oscillators of the molecular clock and the newly discovered components are also necessary, because most of them are transcriptional factors or cofactors, which directly or indirectly regulate a large number of target genes in individual cells, tissues, and organs from dawn to night. To maintain basic growth and development during daily and seasonal cycles, the circadian clock tries to tradeoff between growth and defense by altering circadian pace and robustness of 24-h rhythms. Here, we will elaborate how circadian oscillators actively participate in the stress signaling perception and transduction pathways.

## Environmental and internal *Zeitgebers* in the circadian clock system

Light and temperature, which play roles in plant morphogenesis, are the most important *Zeitgebers* of the circadian clock. Bright light, high temperature, and cold stimuli imposed at different times of a day significantly cause the phase shifts of the circadian rhythms. The forward shifts, backward shifts, or non-response (dead zone) in the plotted phase response curve (PRC) reflects the sensitivity of the circadian clock to the rhythmic entrainment stimulus (Bruce et al., 1960; Pittendrigh, 1960; Johnson, 1999). Bright light pulses usually cause larger phase shifts, up to 11–12 h (defined as “Type 0” PRC) (Salomé and McClung, 2005; Yuan et al., 2020). Another feature of the light pulse PRC is that there is no gradual transition between maximum phase advance and maximum phase delay, showing a big jump. However, phase shift caused by external high temperature or low temperature treatment is usually about 4–6 h. Compared to light pulse PRC, temperature pulses cause smaller phase shifts (defined as “Type 1” PRC) (Johnson, 1999; Xu et al., 2022a).

In addition, the light intensity is inversely related to the period length of circadian rhythm, in other words, the higher the light fluence, the shorter the period length (Xu et al., 2022b). It is known that blue or red light treatment can change the period length of *CAB2:LUC* rhythm, and phytochromes and cryptochromes play critical roles in the entrainment of the circadian clock (Somers et al., 1998). Based on the analysis of effect of light fluence on the period length, phytochrome B acts as a high-intensity red light photoreceptor in circadian input pathway, and phytochrome A acts under dim red light. In the test of blue light regime, it was found that both cryptochrome 1 and phytochrome A act to transmit low-fluence blue light cue to the clock, while cryptochrome 1 and cryptochrome 2 mediate the high-fluence blue light for circadian period control (Somers et al., 1998). Moreover, the ZEITLUPE (LOV-KELCH PROTEIN 1/ADAGIO 1)/LKP (LOV Kelch Protein2)/FKF1 (FLAVIN BINDING, KELCH REPEAT, F-Box 1/LKP3/ADO3) family of proteins in Arabidopsis contain a light-sensing LOV domain that mediates light-fluence-dependent regulation of period length and photomorphogenesis (Millar et al., 1995; Nelson et al., 2000; Somers et al., 2000; Schultz et al., 2001; Imaizumi et al., 2003).

Recent studies have revealed that internal signals act as biochemical cues to reset the circadian clock. The concentration of photosynthetic products, such as glucose and fructose, presents a 24-h circadian oscillation in leaves with the peak of enrichment is around ZT4 in the morning after light exposure (Haydon et al., 2013). The sugar pulse PRC imposed with sucrose and mannitol treatments show that photosynthetic products cause relatively small phase shifts of circadian rhythm, which belong to “Type 1” PRC. When photosynthesis is artificially blocked, the rhythmic oscillation of sugars is significantly blocked, indicating that the relay function of sugar as a light signal serves as the timing cue of the circadian clock. In addition, inorganic nitrogen and nitrogen assimilation products, such as KNO_3_, NH_4_NO_3_, glutamate, and glutamine can also be used as a timing stimulus to reset circadian rhythm at different time of a day, thereby plotting a “Type 1” nitrogen PRC (Gutierrez et al., 2008). It can be concluded that *Zeitgebers* perceived by plants are diverse, and the ambient light, temperature and humidity, internal carbon and nitrogen nutrition, and likely mineral elements in organelles all regulate circadian pace in a hierarchical network (Duc et al., 2009; Hermans et al., 2010; de Melo et al., 2021). The drastic fluctuations in environmental signals, such as high light and high temperature stress at noon, low temperature or even freezing at night, water and nutrients supply deficiency in the air and soil, will inevitably occur during the course of life in land plants. Therefore, to maintain rhythmic growth, cell differentiation, photoperiodic flowering, tuberization, leaf senescence, and seasonal dormancy, the circadian clock spatiotemporally fine-tunes the rhythmic activities and energy allocation to acquire resistance to daily and seasonal stresses (Fig. 1).

## Multiple TTFLs of the molecular clock

The core of Arabidopsis circadian clock is composed of interlocked transcription-translation feedback loops (TTFLs), which maintain the clock pace of nearly 24 h (Hsu and Harmer, 2014) (Fig. 1). CIRCADIAN CLOCK ASSOCIATED 1 (CCA1) and LATE ELONGATED HYPOCOTYL (LHY), two homologous MYB-like transcription factors expressed around dawn, inhibit the expression of *PSEUDO-RESPONSE REGULATORs* (*PRR9*, *7*, *5*, *3*, *1*) and evening-phased *EARLY FLOWERING 3* (*ELF3*), *ELF4*, and *LUX ARRHYTHMO* (*LUX*), which form a ELF3-ELF4-LUX evening complex (EC) (Lau et al., 2011; Nagel et al., 2015; Kamioka et al., 2016). In turn, PRR9, 7, 5, and TIMING OF CAB EXPRESSION 1 (TOC1/PRR1) were sequentially expressed throughout the daytime and repress the transcription of *CCA1* and *LHY* (Nakamichi et al., 2005; Nakamichi et al., 2010; Huang et al., 2012). PRR3 is a tissue-specific regulator and functions in vasculature (Para et al., 2007). The transcription of *PRR9* and *PRR7* is also negatively regulated by the EC complex (Huang et al., 2016). In addition, LIGHT-REGULATED WD1 (LWD1) interacts with TCP transcription factor to form a LWD1-TCP20 complex that activates the transcription of *CCA1* in early morning (Wu et al., 2008; Wu et al., 2016). When TCP21, also known as CCA1 HIKING EXPEDITION (CHE), interacts with TOC1, its inhibition of the *CCA1* transcription can be eliminated (Pruneda-Paz et al., 2009; Gendron et al., 2012). It was recently found that zinc finger transcription factors BBX18 and 19 dynamically recruit PRR9, 7, 5 to the promoter regions of *CCA1*, *LHY*, and *RVE8*, and act as negative regulators in the daytime to repress the expression of morning-phased clock genes (Yuan et al., 2021b).

The evening-phased repressor TOPLESS interacts with PRR9, 7, 5 and histone deacetylases (HDA6) to negatively regulate the expression of *CCA1* (Wang et al., 2013). REVEILLE 4 (RVE4) and RVE8, MYB-LIKE factors that are expressed in the morning interact with cofactor NIGHT LIGHT-INDUCIBLE AND CLOCK-REGULATED 1 (LNK1) and LNK2 to form the morning complex and promote the expression of *PRR5* and *TOC1* (Xie et al., 2014). LNK1 recruits RNA polymerase II and transcription elongation factor to its target genes and affects the deposition of Tri-methylation of lysine 4 on histone H3 (H3K4me3) or chromatin modification on *PRR5* and *TOC1* (Ma et al., 2018). In molecular architecture of the circadian clock, core oscillators also undergo post-transcriptional modifications. Epigenetic mechanisms such as genome DNA methylation, histone deacetylation, and alternative splicing of pre-mRNA affect the expression of circadian clock genes (Ni et al., 2009; Sanchez et al., 2010; Wang et al., 2013; Seo and Mas, 2014). In addition, proteolysis and turnover of core oscillators based on phosphorylation or O-glycosylation modification are also the result of diurnal adaptation (Fujiwara et al., 2008; Wang et al., 2010; Wang et al., 2020a; Yan et al., 2021). Recently, ELF3 has been found to undergo phase separation and function as a thermosensor in response to temperature changes (Jung et al., 2020). In this article, we will continue to summarize the post-transcriptional regulation in different types of circadian gating of stress responses.

## Zeitnehmer ELF3 mediates circadian gating of both light and temperature responses

Most of the core oscillators and circadian-related components involved in the molecular architecture of TTFLs respond to numerous environmental cues, including stress signaling (Harmer et al., 2000; Grundy et al., 2015; Seo and Mas, 2015; Markham and Greenham, 2021). In this review, we summarized the phenotypes of the circadian clock gene mutants under abiotic stresses (Table 1). Among them, ELF3 is regarded as a key factor in light resetting, high temperature response, and salinity tolerance of the circadian clock (McWatters et al., 2000; Covington et al., 2001; Carré, 2002; Thines and Harmon, 2010; Box et al., 2015). *ELF3* was first discovered to act on the regulation of flowering time, and loss-of-function of *ELF3* in *elf3* alters the photoperiodic induction of flowering and diurnal control of hypocotyl growth in Arabidopsis and crops (Zagotta et al., 1996; Boden et al., 2014; Sakuraba et al., 2016; Lu et al., 2017; Jiang et al., 2019). In subsequent studies, it was found that *ELF3* was intensively expressed at night, and *elf3* null mutant showed an obvious light-dependent arrhythmia of circadian output (Covington et al., 2001). The altered *ELF3* amount in *elf3* and *ELF3-OX* overexpression lines significantly affect the phase shifts of strong red and blue light pulses to the circadian rhythm (Covington et al., 2001; Thines and Harmon, 2010). Therefore, ELF3 guards light resetting or sensitivity of the circadian clock to light cue.

**Table 1:** Physiological phenotypes of circadian clock gene mutants under abiotic stress conditions

Clock Mutants	Species*	Physiological Phenotype	Literatures
MYB-like transcription factors			
*cca1 lhy*	Arabidopsis	Reduced freezing tolerance; Sensitive to ambient iron level; Hypocotyl elongation is less sensitive to higher ambient temperature	Dong et al., 2011; Kidokoro et al., 2021; Sun et al., 2019; Xu et al., 2019
*CCA1-ox*	Arabidopsis	Reduced sensitivity to iron deficiency	Xu et al., 2019
*rve4 rve8*	Arabidopsis	Reduced tolerance to freezing and high temperature	Kidokoro et al., 2021; Li et al., 2019
*rve1 rve2*	Arabidopsis	Enhanced tolerance to freezing	Kidokoro et al., 2021
*gmlclq*	Soybean	Increased tolerance to freezing	Wang et al., 2021; Yuan et al., 2021b
Pseudoresponse regulators (PRRs)			
*prr9 prr7 prr5*	Arabidopsis	Reduced leaf dehydration; Enhanced tolerance to low temperature, salinity and drought stress	Liu et al., 2013; Nakamichi et al., 2009
*PRR7-ox*	Arabidopsis	Increased water loss from leaf; Sensitive to iron excess	Liu et al., 2013
*PRR5-ox*	Arabidopsis	Hypocotyl elongation is less sensitive to high temperature	Zhu et al., 2016
*TOC1-ox*	Arabidopsis	Reduced tolerance to drought; Hypocotyl elongation is less sensitive to higher ambient temperature	Legnaioli et al., 2009; Zhu et al., 2016
*TOC1 RNAi*	Arabidopsis	Increased tolerance to drought stress	Legnaioli et al., 2009
*TOC1 RNAi*	Tobacco	Reduced leaf dehydration	Valim et al., 2019
*osprr73*	Rice	Reduced tolerance to high salinity	Wei et al., 2021
*ppd-H1*	Barley	More sensitive to mild drought stress	Gol et al., 2021
Evening Complex			
*ELF3-ox*	Arabidopsis	Enhanced tolerance to high salinity	Sakuraba et al., 2017
*elf3–1, lux-4*	Arabidopsis	Growth rate is not sensitive to high temperature	Box et al., 2015
*j* (*AtELF3* homolog)	Soybean	Reduced tolerance to high salinity	Cheng et al., 2020
GIGANTEA (GI)			
*gi-3*	Arabidopsis	Reduced tolerance to freezing	Cao et al., 2005
*gi-2*	Arabidopsis	Reduced tolerance to drought stress	Baek et al., 2020
*gi-1*	Arabidopsis	Enhanced tolerance to high salinity	Kim et al., 2013; Sakuraba et al., 2017
*GI-ox*	Arabidopsis	More sensitive to high salinity	Kim et al., 2013
*gi-1*, gi*-3*	Chinese cabbage	Enhanced tolerance to freezing	Xie et al., 2015
ZEITLUPE (ZTL)			
*ztl-105*	Arabidopsis	Reduced tolerance to high-temperature stress	Gil et al., 2017
*ZTL-ox*	Arabidopsis	Enhanced tolerance to high-temperature stress	Gil et al., 2017
TIME FOR COFFEE (TIC)			
*tic-2*	Arabidopsis	More sensitive to ambient iron level	Duc et al., 2009

*Arabidopsis (*Arabidopsis thaliana*); Soybean (*Glycine max*); Tobacco (*Nicotiana attenuate*); Rice (*Oryza sativa*); Barley (*Hordeum vulgare*); Chinese cabbage (*Brassica rapa*)

Moreover, ELF3 is also required for the circadian clock in the responses to ambient temperature fluctuations (Thines and Harmon, 2010). The binding of evening-phased ELF3 and EC complex to the promoters of their target genes is temperature-dependent, and ELF3 and LUX regulate the expression of *LNK1* under cold- or warm-night by directly binding to *LNK1* promoter (Mizuno et al., 2014; Box et al., 2015). BBX18 interacts with ELF3 in thermomorphogenesis, and BBX18 together with E3 ligase XBAT31 and XBAT35 promote the degradation of ELF3 protein during warm night (Ding et al., 2018; Zhang et al., 2021a; Zhang et al., 2021b). Moreover, temperature directly controls ELF3 activity through the mechanism of protein aggregation (Jung et al., 2020). When the ambient temperature is maintained at 22 °C, ELF3 proteins diffuse, and when the temperature rises to 27 °C, ELF3 undergoes phase separation and forms protein speckles. ELF3 in crop is involved in the regulation of environmental adaptation. In short-day soybean genetic resources introduced from north to south, the delay in flowering and maturity caused by the loss-of-function of *J*, *AtELF3* homolog, is conducive to increasing the yield of soybean in low latitudes (Lu et al., 2017). In summary, the circadian core oscillators are regulated by environmental stimuli at transcriptional and post-transcriptional levels. In addition, the circadian clock drives the self-sustaining 24-h rhythms over a broad range of physiological temperatures, a characteristic termed temperature compensation, but the underlying mechanism are largely unknown.

## Circadian gating of plant thermomorphogenesis

Pittendrigh discovered the temperature independence in the clock system (Pittendrigh, 1954). Further results show that circadian period length is maintained at about 24 h over a broad range of physiological temperatures, suggesting “temperature compensation” is the core property of the circadian clocks (Pittendrigh, 1954; Zimmerman et al., 1968; Edwards et al., 2005; Xu et al., 2010). Edwards et al. completed the QTL mapping of the circadian parameters of the leaf movement rhythm at 12 °C, 22 °C, and 27 °C in recombinant inbred lines (RILs) of Arabidopsis accession Columbia (Col) by Landsberg erecta (Ler) and Cape Verde Islands (Cvi) by Ler (Edwards et al., 2005). This study identified circadian clock-controlled and flower-timing gene *GIGANTEA* (*GI*) and an F-box E3 ubiquitin ligase gene *ZEITLUPE* (*ZTL*) as strong candidates for two of the QTLs involving in temperature compensation.

The degradation of PRR5 and TOC1 proteins is mediated by ZTL (Fujiwara et al., 2008). GI stabilizes ZTL protein and facilitates ZTL maturation, and ZTL also contains a blue-light-absorbing LOV (Light, Oxygen, or Voltage) domain, which is critical for ZTL function on circadian rhythms (Kim et al., 2007; Cha et al., 2017; Pudasaini et al., 2017). A recent study found that in Arabidopsis Cvi, an accession line generated in Cape Verde Islands environment, the protein-protein interaction between GI^Cvi^ and ZTL^*Cvi*^ is temperature-dependent, which participates in the accumulation of ZTL protein and maintains a circadian period for nearly 24 h at warm temperatures (27 °C) (Kim et al., 2020). It is also found that ZTL contributes to lengthened circadian period of the root clock (Li et al., 2020b). In *ztl-3* mutant, the difference in period length between roots and shoots was significantly greater than that in wild type, indicating that ZTL may have different effects on the temperature response in multiple plant tissues. In the analysis with 30 °C, the absence of *PRR9* and *PRR7* in *prr7 prr9* double mutant is critical to the temperature-over-compensation (Salomé et al., 2010). Moreover, compared with 22 °C, circadian amplitude of *GI* expression in roots was weakened at 28 °C, and the inhibitory effect of PRR9 and PRR7 on *GI* expression was also reduced in roots under warm temperature (Yuan et al., 2020).

**Fig. 2:**
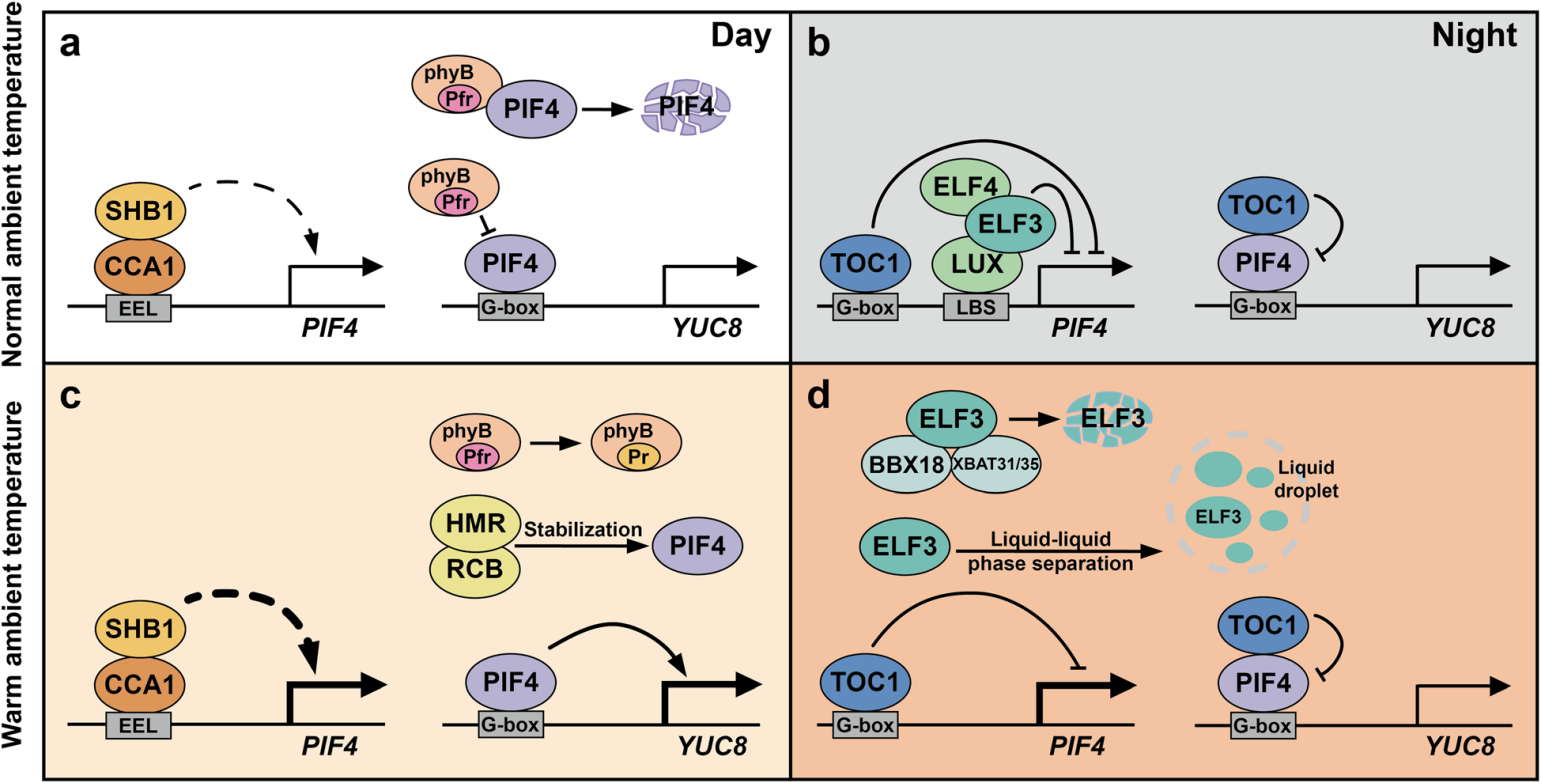
The circadian oscillators regulate the diel expression of *PIF4* in thermomorphogenesis

The rhythmic growth of hypocotyls is jointly regulated by light, ambient temperature and the circadian clock. The sequential expression of *PRR9*, *7*, *5*, and *TOC1* from dawn to early evening dynamically inhibits the hypocotyl elongation in daytime under short-day conditions (Martin et al., 2018). These PRRs interact with bHLH transcription factor PHYTOCHROME INTERACTING FACTOR (PIF3, 4) proteins and restrict PIFs activity in the morning (Soy et al., 2016; Zhu et al., 2016; Martin et al., 2018). PRR-PIF complex negatively regulate their common target genes during the daytime, such as PIF-promoted *CDF5*, which promote cell elongation in the morning (Martin et al., 2018). During the course of the night, TOC1 interacts with PIF4, thereby inhibiting the activation of PIF4 on its target genes. At a warm temperature of 27 °C, PIF4 mediates the increase of phytohormone auxin and promotes the elongation of hypocotyls in Arabidopsis (Gray et al., 1998; Koini et al., 2009; Franklin et al., 2011) (Fig. 2). Moreover, warm temperature specifically accelerates the rapid growth of hypocotyls at nighttime, and this common feature of hypocotyl thermo-responsiveness shows natural variation in Arabidopsis ecotypes (Box et al., 2015). In the analysis with the multiple natural accessions, the temperature-dependent expression of evening-phased gene *ELF3* and *LUX* weakened in specific lines with faster hypocotyl growth rate at nighttime. The accumulation of *PIF4* transcripts at 22 °C and 27 °C is largely affected by the functional expression of *ELF3*. Compared with wild type and *lux-4*, *elf3–1* mutant lost thermal responsiveness, indicating that evening components are required for circadian gating of thermoresponsive growth (Box et al., 2015).

Phytochrome B (phyB) and PIFs have been shown to both preferentially target G-box motifs in promotor regions of target genes. In addition to ELF3, phyB, which acts as red-light receptor in seedlings de-etiolation, also functions as a thermosensor (Jung et al., 2016; Legris et al., 2016). The rate of phyB inactivation is proportional to temperature under dark conditions. Therefore, phyB senses the temperature fluctuations and directly inhibits its target genes during the night. In the analysis of RNA-seq and ChIP-seq, it was also found that the ability of EC complex and phyB to bind target genome-wide depends on temperature (Ezer et al., 2017). This study suggests that evening-phased circadian oscillators and the phyB jointly mediate the transduction of temperature cues (Fig. 2). It was recently discovered that hypocotyl elongation of *cca1 lhy* mutant is not sensitive to warm temperature of 29 °C (Sun et al., 2019). Morning-phased CCA1 recruits SHORT HYPOCOTYL UNDER BLUE1 (SHB1) to *PIF4* promoter and mediates red light-induced *PIF4* expression, thereby maintaining the light response and warm temperature morphogenesis during the morning (Sun et al., 2019) (Fig. 2). The recently identified HMR (HEMERA)-RCB (REGULATOR OF CHLOROPLAST BIOGENESIS) protein complex promotes the accumulation of PIF4 to initiate thermomorphogenesis during daytime (Qiu et al., 2021).

## Coordination of circadian timing in high-temperature tolerance

Gil et al. found that overexpression of *ZTL* significantly improved the acquisition of thermotolerance of seedlings, and the survival rate was close to 100% after exposure to heat shock at 45 °C for 1.5 h in darkness (Gil et al., 2017). The exposure to 40 °C for 4.5 h in darkness at the midday greatly reduced the acquired thermotolerance in *ztl-105* mutant with only a 10% survival rate, compared with the wild-type survival rate of 60%, and nearly 95% of *ZTL* overexpression line that experienced heat shock survived. In the analysis of total proteins using an anti-ubiquitin antibody, the amount of polyubiquitinated proteins (Ub)_n_ in heat-shocked *ztl-105* were reduced by about 30% compared with that in the wild-type plants. In addition, heat shock-induced polyubiquitination is dependent on the expression of HSP90 and the protein-protein interaction between ZTL and HSP90. Analysis with high-resolution nano-LC-MS shows that ZTL is involved in protein quality control system and is responsible for the removal of high-temperature-induced harmful protein aggregates (Gil et al., 2017).

The survival rate of *toc1 prr5* double mutant subjected to a 45 °C heat shock is much higher than that of wild type and *TOC1* overexpression plant, indicating that the rhythmic growth controlled by evening gene *TOC1* is essential for high temperature tolerance (Zhu et al., 2016). The expression of *PIF4* and its target gene *YUC8* were significantly promoted at ZT8–16 and ZT16–20 in *toc1* and *toc1 prr5* mutants, suggesting the inhibitory effects of evening-accumulated TOC1 on *PIF4* expression at late afternoon and evening during thermoresponsive growth (Zhu et al., 2016) (Fig. 2). During nighttime, TOC1 and ELF3-ELF4-LUX bind to the G-box or LBS element of *PIF4* promoter, respectively, to inhibit *PIF4* expression (Nusinow et al., 2011). In addition, based on transcriptional profiling, *RVE4* and *RVE8* play a role in the early response to heat shock signaling (Li et al., 2019).

The tissue-specific expression and communication of circadian oscillators have also been studied (Endo et al., 2014; Takahashi et al., 2015; Li et al., 2020b). The circadian clocks in shoots and roots can be independently entrained by imposed light and temperature stimuli (Thain et al., 2000; Wang et al., 2020b). In the tissues of the shoot apex, vasculature, root, and root tip, the cell-autonomous circadian clock effectively controls the period length of the local rhythms such as transcript rhythmic accumulation and dynamic protein-protein interactions (Gould et al., 2018; Li et al., 2020b). The circadian clock of shoots and roots independently respond to high temperature pulses, and the circadian rhythms in both tissues have the features of temperature compensation (Li et al., 2020b). Compared to shoots, the rhythmic expression of *PRR9*, *PRR7* and EC complex genes in roots is more sensitive to the increase of environmental temperature (Yuan et al., 2020). Recently, grafting experiment confirmed the movement of ELF4 protein from shoot to root, and the ELF4 transport was inhibited by high temperature (Chen et al., 2020). As a result, the accumulation of ELF4 protein in the roots under high temperature was reduced, which may further promote the expression of *PRR9*, resulting in an increase in circadian pace of the root clock. Moreover, in field-grown rice panicles, warm nighttime temperatures increase *OsPRR95* expression and disrupt the circadian-associated targets based on the transcriptome analyses (Desai et al., 2021).

In short, these studies show that the clock components closely co-occur with the light and temperature signaling and accurately regulate the light and temperature-dependent growth during the day and night. Among them, multiple circadian core oscillators may also involve in plant temperature tolerance in response to climate changes.

## Daily and seasonal cold acclimation and freezing tolerance

Since they are rooted into the ground and cannot move, plants have to undergo frigid temperatures and cold acclimation to enhance freezing tolerance, especially in winter. A series of studies have shown that low temperature affects the circadian rhythm and the transcription of core oscillators in Arabidopsis. For example, with the increase in the number of days of cold treatment (4 °C), the robustness of the free-running rhythm in constant light of clock gene transcripts becomes weaker or dampened compared to 20 °C (Bieniawska et al., 2008). In addition, pre-mRNA of *CCA1* undergoes alternative splicing (AS), which is inhibited by cold temperatures (Filichkin and Mockler, 2012; Seo et al., 2012). The retention of intron and splicing of alternative exon in most clock genes are associated with the temperature-dependent AS. It is found that the cold temperature of 4 °C significantly alters the production of *CCA1*, *LHY*, and *PRRs* transcript isoforms (James et al., 2012). Seo et al. has found that cold treatment increases the *CCA1α* isoform amount and reduces the *CCA1β* isoform accumulation (Seo et al., 2012). Overexpression of *CCA1α* causes an increase in *CBF*s expression, thus enhancing the freezing resistance of plants. The hypothesis proposed in this study is that CCA1β mediates the response of CCA1 to low temperature by interfering with the formation of CCA1α-CCA1α, LHY-LHY homodimers, and CCA1α-LHY heterodimers.

In winter freezing tolerance of populus tree, the shoot apical meristem remains dormant and buds burst in early spring. When the expression of clock genes is knocked down by RNAi, the tree shows a faster clock pace of the circadian rhythm, and its acclimation to freezing is remarkably weakened (Ibanez et al., 2010). In two assessments of electrolyte leakage (− 18 °C to nearly − 70 °C) and tissue discoloration (− 18 °C and − 51 °C) to determine injury, the reduction in freezing tolerance in *pttlhy* mutant (down-regulation of *PttLHY1* and *PttLHY2*) was significant compared to the wild type. Moreover, loss-of-function of *PttLHY* or *PttTOC1* causes delayed growth cessation during the shift from 18-h light exposure of photoperiod to 15-h light exposure, indicating that the mutants lost the photoperiodic sensitivity to critical daylength. In further experiments, bud burst of *pttlhy* mutant was also significantly delayed when shifting from a short-day to a long-day photoperiod. All in all, this study confirmed that functional circadian oscillators are critical to photoperiodism and low temperature resistance of plants.

In addition to the greatly reduced *PttLHY* transcripts in early morning, in *pttlhy* mutant, the peak phase of *FKF1* and *GI* rhythmic expression was largely shifted forward compared to wild type (Ibanez et al., 2010). In the mechanism of photoperiod flowering, FKF1 and GI form a complex to degrade CDF protein, which removes the repressor from inhibiting *CO* and *FT* gene transcription (Imaizumi et al., 2005; Fornara et al., 2015). FKF1and ZTL transcripts accumulation are controlled by the circadian clock. These two proteins belong to another class of blue-light photoreceptor and both own a unique structure containing a PAS domain at N-terminus, an F-box and kelch repeats at C-terminus (Nelson et al., 2000; Somers et al., 2000). ZTL and FKF1 regulate flowering time specifically in response to long days. Interestingly, the late flowering phenotype of *fkf1* mutant was restored by vernalization under 4 °C in dim white light for 4 weeks (Nelson et al., 2000). This result suggests that FKF1 plays a role in freezing tolerance by regulating cold acclimation of plants.

**Fig. 3:**
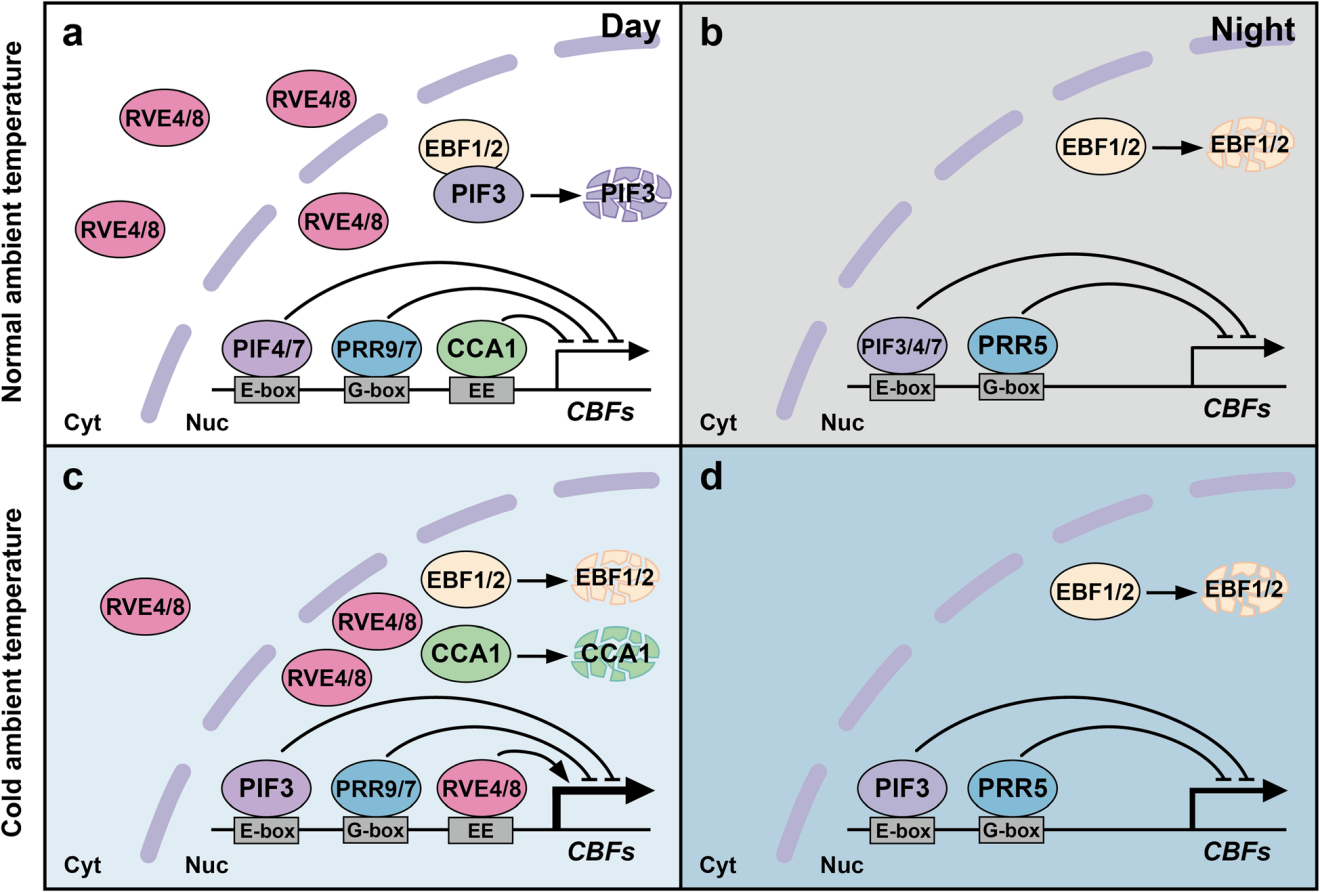
MYB-like transcription factors and PRRs in the circadian clock regulate the transcription of *CBFs* in response to low temperatures

In Arabidopsis, many circadian core oscillators are involved in low-temperature signaling (Fig. 3). It is found that *prr9–11 prr7–10 prr5–10* triple mutant (*d975*) and *amiR-PRR9-PRR7* line are more tolerant to cold and freezing stress than the wild type (Nakamichi et al., 2009; Wang et al., 2017). Both *cca1 lhy* and *rve4 rve8* double mutants display a freezing-sensitive phenotype (Dong et al., 2011; Kidokoro et al., 2021). As MYB-like transcription factors, CCA1 and LHY occur closely with RVE4 and RVE8 in the early morning. CCA1 and LHY act as transcription repressors in the molecular architecture, while RVE4 and RVE8 act as activators. In response to low temperature, their loss-of-function mutants also shows different effects on cold signaling. Using the analysis of subcellular localization of the protein-labeled GFP, it is found that, after cold stress treatment for 3 h, the fluorescence of CCA1, LHY protein has completely disappeared, while RVE4 and RVE8 accumulated in the nucleus specifically upon cold stress (Kidokoro et al., 2021). ChIP-qPCR result confirms that under low temperature, the binding of CCA1 and LHY is reduced, while the RVE4 and RVE8 binding is increased. From this point of view, RVEs positively regulated the target genes, and CCA1 and LHY may have an indirect effect on the regulation of cold induction of C-repeat Binding Factor gene *CBF1*, *CBF2*, and *CBF3* (Dong et al., 2011; Kidokoro et al., 2021). What’s worth thinking about is that in *cca1–11 lhy-21* double mutant, the circadian amplitude of *CBF1*, *CBF2*, and *CBF3* (also known as *DROUGHT RESPONSE ELEMENT BINDING FACTOR 1B*, *1C*, and *1A*) or CBF-targeted cold-responsive gene (*COR*s) expression (*COR15A*, *COR47*, and *COR78*) is significantly reduced under cold temperature (4 °C) acclimation (Dong et al., 2011). This result predicts that CCA1 and LHY may not directly regulate the transcription of genes related to cold acclimation.

It has been reported that PRR9, PRR7 and PRR5 inhibit the expression of *CBFs* during the day and night, respectively, by binding to G-box elements (Liu et al., 2016). In addition, the binding of PIF3, 4, and 7 to E-box element inhibits the expression of *CBFs* during the day (Lee and Thomashow, 2012; Kidokoro et al., 2021). Under low temperature conditions, the stability of EBF1, 2 (EIN3-BINDING F-BOX) decreases, so its function on degradation of PIF3 is weakened (Jiang et al., 2017) (Fig. 3). Wang et al. found that *COR27* and *COR28*, which are regulated by the circadian clock with peak phase occurs at night, are genetically related to *PRR9* and *PRR7* and jointly contribute to the resistance to freezing (− 7 °C) without prior cold acclimation (Wang et al., 2017). The results of chromatin immunoprecipitation show that the directly binding of CCA1 to the promoters of *COR27* and *COR28* were attenuated under cold temperature conditions. In summary, the expression of circadian genes and the stability of post-transcriptional clock proteins are sensitive to temperature fluctuations during the day or night.

The clock components identified under extreme low temperature conditions schedule multiple signaling pathways, including circadian rhythms. In *d975* plants, circadian components, such as *CCA1*, *TOC1* and *GI*, are expressed arrhythmically (Nakamichi et al., 2005). The *d975* mutant is actually resistant to a variety of stresses such as cold, high salinity, and drought (Nakamichi et al., 2009). It is known that *gi-3* plant shows an increased sensitivity to freezing stress (Cao et al., 2005). Moreover, genetic analysis in *Brassica rapa* shows that natural variation in *GI* is responsible for flowering, circadian period length, freezing resistance, and salt tolerance (Xie et al., 2015). Taken together, the circadian oscillators are associated to daily and seasonal low temperature responses.

## Circadian clock gates ABA signaling in response to drought stress

**Fig. 4:**
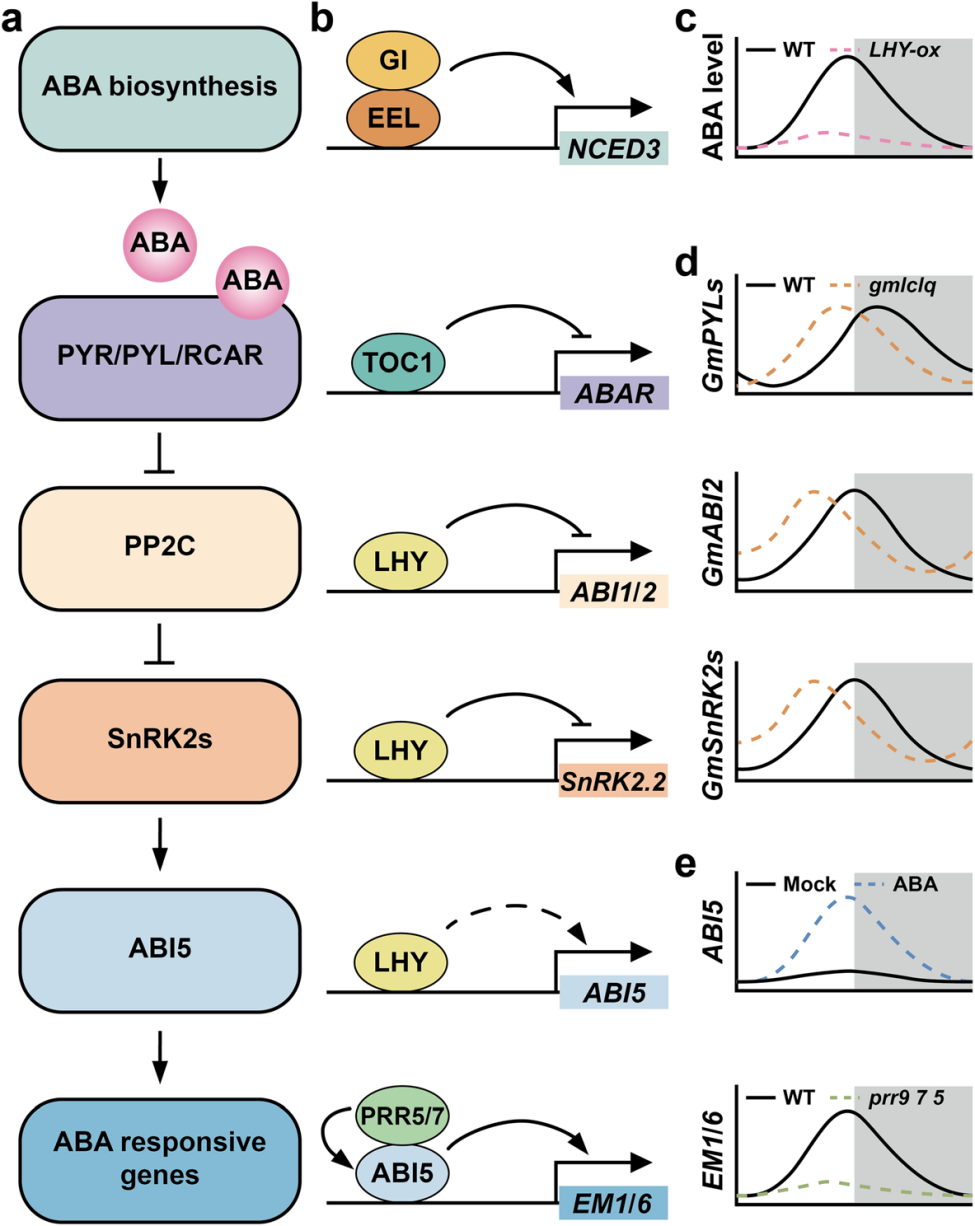
Circadian clock gates ABA signaling in response to drought stress

The circadian clock gates plenty of hormonal cross-talks, including hormone biosynthesis, degradation, and signaling pathways (Hanano et al., 2006; Covington et al., 2008; Atamian and Harmer, 2016). Mockler et al. established diurnal and circadian expression profiling in the diurnal project (DIURNAL database, http://diurnal.cgrb.oregonstate.edu/), which is widely used to retrieve a large number of clock-controlled genes that regulate hormone metabolism, perception and responses in Arabidopsis and crops (Mockler et al., 2007). Numerous hormones are involved in the plant resistance to various abiotic and biotic stresses (Zhu, 2016). Based on the established time course transcriptome analysis, it was found that transcript enrichment of a large number of abscisic acid (ABA)- and methyl jasmonate (MJ)-responsive genes show rhythmic oscillation within a 24-h cycles (Mizuno and Yamashino, 2008). These two types of hormones constitute the most basic signaling network for plant growth and stress resistance, thus indicating that the circadian clock contributes to plant adaption to ambient stresses (Fig. 4).

Take drought stress as an example, when plants are faced with water deficiency, ABA accumulates and induces ABA-mediated drought responses, like the promotion of stomatal closure to reduce water loss. During day and night cycles, the ABA level in leaves oscillates robustly, accumulating during the day, reaching its maximum level around noon, then gradually decreasing to the lowest level before dawn (Lee et al., 2006). This ABA accumulation rhythm is completely synchronized with the diurnal fluctuation of transpiration rate, suggesting that ABA likely acts as an internal cyclic signal to cue water level over a 24-h day (Fig. 4c). Leaf dehydration increases the ABA-GE hydrolyzing activity of β-glucosidase, and the expression of *AtBG1* gene encoding β-glucosidase dynamically determines ABA production and its diurnal oscillation. The *AtBG1* mutation results in a defect in stomatal closure in ABA responses. The peak expression of ABA-induced genes mostly occurs during daytime, displaying circadian rhythmicity (Mizuno and Yamashino, 2008). All of the above indicate that the circadian clock gates ABA signaling in response to drought stress. In turn, ABA level fluctuations also influence the endogenous circadian rhythms. The exogenous application of ABA results in a slight shortening in the period length of circadian rhythm (Liu et al., 2013). In addition, ABA-treated *prr* mutants display more sensitive phenotype than wild-type and *d975* triple plant, that is, showing stronger reduction in period length shortening and the remarkable increased water loss rate in leaves. This information indicates that the ABA gives feedback to the circadian clock and endogenous 24-h rhythms.

Functional circadian oscillators are necessary for drought responses (Fig. 4). Overexpression of *TOC1* significantly blocks the tolerance to drought (Legnaioli et al., 2009). In plants overexpressing *TOC1*, leaves were severely dehydrated, the stomata closure was no longer sensitive to ABA treatment, and the stomata conductance was significantly higher than that of the wild type, thus reducing the survival rate under dehydration stress. ChIP analysis revealed that *TOC1* overexpression inhibits ABA-related *ABAR* gene transcription possibly by directly binding to the *ABAR* promoter. Exogenous application of ABA triggers TOC1 to bind to the *ABAR* promoter more during the day than untreated materials and inhibit the rhythmic enrichment of *ABAR* in daytime (Legnaioli et al., 2009). The contribution of tissue-specific circadian clock has also been found in drought tolerance studies. *TOC1* functions in tobacco (*Nicotiana attenuate*) in response to drought were investigated separately in shoots and roots. (Valim et al., 2019). Under long-term drought field conditions, comparing root-only *TOC1*-deficient plant with wild type, *TOC1* function in shoots is sufficient for wild-type allometric drought responses. Moreover, co-expression analysis in whole-transcriptome microarray profiling links the function of shoot *TOC1* with red and far-red light signaling to drought responses.

Overexpression of *PRR7* causes faster water loss rate in leaves, while in *d975* triple mutant, dysfunction of *PRR9*,*7*,*5* significantly reduces stomata conductance and leaf water loss rate, thereby enhancing the tolerance to drought stress (Nakamichi et al., 2009; Liu et al., 2013). Furthermore, in *d975* plant treated with 10 μM ABA, the expression of ABA biosynthetic gene *ABA1* and ABA-induced cycling DOF family gene *CDF1* is increased, compared with the wild type treated with exogenous ABA (Liu et al., 2013). Therefore, PRRs core oscillators act as transcription repressors to negatively regulate the ABA response and signal transmission. Barley (*Hordeum vulgare*) photoperiod response gene *PHOTOPERIOD-H1* (*Ppd-H1*) is a homolog to Arabidopsis *PRR*. Gol et al. found that mild drought causes a decrease in the number of spikelet and a delay in floral development in spring barley cultivars with a natural mutation in *ppd-H1*, but has little effect on the introgression lines carrying wild-type *Ppd-H1* (Gol et al., 2021). This result suggests the critical role of circadian component PRRs in potential drought adaptations.

During water deficiency treatment, the *LHY* mutation significantly induces a large amount of ABA accumulation during the day and enhances the robustness of the rhythm of ABA level (Adams et al., 2018). When ChIP-seq analysis was applied to identify the targets of LHY, it was revealed that LHY likely directly binds to and inhibits and multiple target genes of ABA signaling pathways (Adams et al., 2018). The clock-controlled *NCED3* gene encodes a *9-cis*-epoxycarotenoid dioxygenase, which catalyze the rate-limiting step of ABA biosynthesis. LHY binds to promoters of protein phosphatase 2C genes *ABI1*, *2* and *SnRK2.2* to inhibit their expression. LHY also binds to the *ABI5* promoter to directly or indirectly promote its expression. In addition, PRR5 and PRR7 interacts with ABI5 to promote the transcriptional activation of ABI5 protein on ABA-responsive genes *EM1* and *EM6* (Yang et al., 2021). Studies in grain crop soybean (*Glycine max*) found that loss-of-function of *GmLCLs* (*GmLCLa1*,*a2*,*b1*,*b2*), four orthologues of Arabidopsis *CCA1* and *LHY*, promote stomatal closure under drought stress and reduce leaf water loss rate, thereby enhancing the drought tolerance in soybean (Wang et al., 2021; Yuan et al., 2021a). Leaf dehydration causes circadian phase of rhythmic expression of *GmLCLs* to shift from early morning to noon for nearly 4 h (Yuan et al., 2021a). In addition, GmLCLs in soybean, as transcription repressors, negatively regulate the circadian rhythmic expression of ABA-responsive genes (Fig. 4d).

In *gi-1* mutant, ABA level is lower than that of wild type, and stomatal closure is weakened during the leaf dehydration, which may accelerate leaf water loss and reduce survival rate during dehydration stress (Baek et al., 2020). In terms of molecular mechanism, GI interacts with bZIP transcription factor ENHANCED EM LEVEL (EEL), and the GI-EEL complex binds to the ABA-responsive element (ABRE) of *NCED3* (Baek et al., 2020). In summary, to synchronize with internal ABA response, the circadian clock is likely to orchestrate ABA biosynthesis to improve the robustness of ABA level rhythm, and to shift the circadian phase of clock and clock-controlled genes rhythmic expression in response to drought stress.

## Circadian regulation of salinity responses and tolerance

The sensitivity of plants to salinity stress is time-dependent in a 24-h light-dark cycle. The application of 200 mM NaCl at nighttime has little effect on plant growth based on measurement of shoot fresh weight, while the daytime application causes a significant inhibitory effect (Park et al., 2016). Dysfunction of circadian oscillators significantly alters the resistance to salt stress. When plants were subjected to 250 mM NaCl treatment for 6 days, the bleaching rate in *d975* triple mutant was only 20%, compared with 80% in wild type, indicating the contribution of PRRs in high salinity tolerance (Nakamichi et al., 2009). GI that regulates PRRs clock protein stability undergoes salt-induced degradation, and the resistance of *gi-1* mutant to 150 mM NaCl treatment is stronger than that of wild type (Kim et al., 2013). In addition, overexpression of *ELF3* significantly improves the resistance to 200 mM NaCl, and the ion leakage rate in *ELF3-OX* is much lower than that of wild-type plants (Sakuraba et al., 2017).

**Fig. 5:**
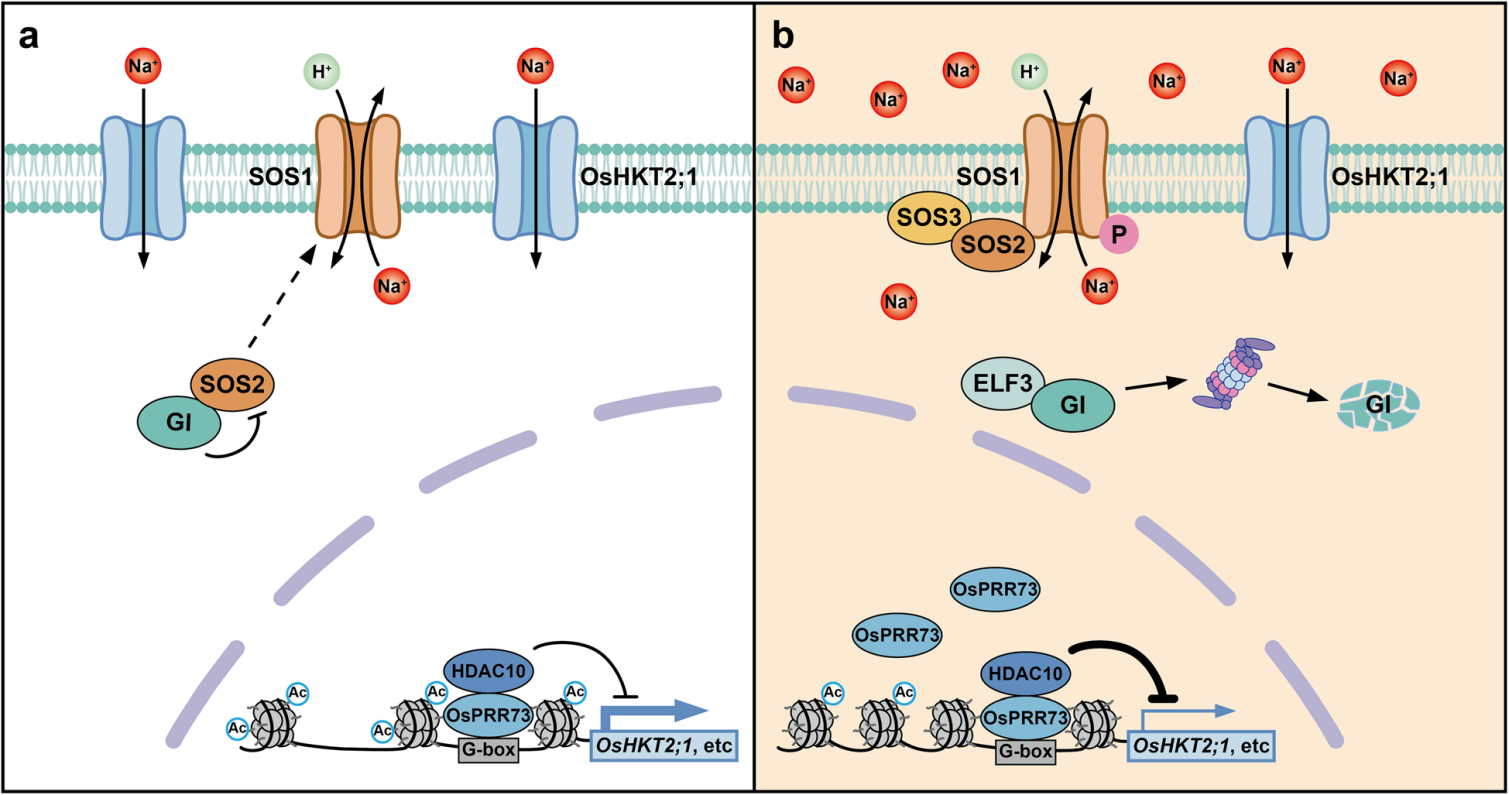
PRRs and GI regulate Na^+^ transport through transcription and post-transcriptional mechanisms, respectively

In terms of molecular mechanism, the circadian clock is closely related to the Salt-Overly Sensitive (SOS) signaling pathway (Zhu et al., 2010; Zhu, 2016; Yang and Guo, 2018). Under non-stress conditions, GI interacts with SOS2 to inhibit its function (Kim et al., 2013). Salt induces the degradation of GI protein, so the released SOS2 is recruited to the plasma membrane to activate SOS1 Na^+^/H^+^ antiporter activity, which extrudes Na^+^ out of the cell (Fig. 5). GI and ZTL interact to form a complex during the day, indirectly enhancing the stability of PRR5 and TOC1 proteins. If the salt stress in the daytime induces GI degradation, the cell-autonomous circadian clock will have serious functional defects. Genetic mapping and analysis of heterogeneous inbred lines show that nucleotide polymorphism of *Brassica rapa GI* is responsible for variation in circadian period, salt tolerance, cold response, and red light-mediated photomorphogenesis (Xie et al., 2015). Overexpression of clock evening complex component ELF3 promotes GI degradation. On the contrary, GI protein level remains higher in *elf3–1* mutant (Sakuraba et al., 2017). At the same time, ELF3 promotes salt stress tolerance by repressing PIF4. PIF4 directly inhibits *ANAC042* gene transcription to modulate plant growth via hormone GA and BR signaling. Also, PIF4 likely upregulates the positive aging regulator gene *ANAC092*/*ORESARA1* (*ORE1*) and senescence-associated gene *SAG29* in response to salinity stress (Sakuraba et al., 2017). Studies in soybeans have confirmed the contribution of *ELF3* ortholog *J* in salt tolerance. The transcriptome data shows that the expression of salt-responsive genes decreases in *j* loss-of-function alleles, indicating the positive regulation of *J* in salt stress resistance (Cheng et al., 2020). In summary, GI, ZTL, and ELF3 are required in molecular mechanisms for plant to orchestrate growth, senescence, and stress resistance.

Epigenetic modification of clock components is involved in the salinity-associated mechanism. It has recently been discovered that *OsPRR73* in rice (*Oryza sativa*) contributes to salt tolerance (Wei et al., 2021). *OsHKT2;1* was identified as the target gene of OsPRR73 by RNAseq, encoding a plasma membrane-localized Na^+^ transporter. Salt-induced expression of OSPRR73 interacts with histone deacetylase 10 (HDAC10) to alter promoter chromatin status of *OsHKT2;1*, thereby inhibiting *OsHKT2;1* transcription (Fig. 5).

All in all, the circadian clock actively mediates the transduction of salt stress signal and physiological regulation of resistance. The rhythmic expression of core oscillators is sensitive to salt signals, and fine-tune the robustness/amplitude, circadian pace, and circadian phase of endogenous 24-h rhythm to activate the salt tolerance.

## The circadian clock perceives mineral nutrition fluctuations and malnutrition

The concentration of cytosolic and chloroplastic free calcium (Ca) and magnesium (Mg) in cells oscillate in a 24-h day, indicating that ions homeostasis is regulated by the circadian clock (Johnson et al., 1995; Sai and Johnson, 1999; Feeney et al., 2016). In Arabidopsis, circadian rhythm of cytosolic free calcium ion ([Ca^2+^]_cyt_) is synchronized with photoperiodic cue, and circadian peak phase occurs in daytime (Love et al., 2004; Xu et al., 2007). Plants need a variety of mineral elements for growth and development, which are transported, stored and utilized in tissues after being absorbed as metal ions from the environment. The destruction of mineral homeostasis triggers an alternation in circadian rhythms. For example, application of 10 μM CuSO_4_ causes a shortened circadian period under constant light or constant darkness and a weakened amplitude of *CCA1* transcripts enrichment rhythm (Andres-Colas et al., 2010). The transcript accumulation of *CCA1* and *LHY* in ZT0 decreases significantly under the growth conditions with 0.2, 1, 10, or 50 mM CuSO4 treatment. These results indicate that disorder of Cu homeostasis represses *CCA1* and *LHY* expression in early morning.

In circadian phase shift assay, 0.25 μM Fe pulse causes obvious phase delay of *TOC1:LUC* rhythm, indicating that Fe element may act as an internal timing cue to reset the circadian clock (Salomé et al., 2013). The extremely low Fe level of 0.25 μM causes leaf chlorosis and slower circadian pace, showing a period length longer than 27 h. The circadian rhythms were further monitored under various Fe level of 0.25, 1, 5, and 25 μM, and it was found that circadian period length is inversely proportional to Fe level. It is noteworthy that, under long-term Fe deficient conditions, circadian rhythms of morning-phased *CCA1*, *LHY*, *PRR9*, and *PRR7* expression are dampened with gradually reduced amplitude, while circadian rhythms of afternoon- and evening-phased *PRR5*, *TOC1* and *ELF3* expression are not obvious altered (Chen et al., 2013). This study suggests that the sensitivity of circadian clock to Fe fluctuations is time-dependent over a 24-h day, and Fe likely fine-tune circadian rhythm via a retrograde signaling pathway.

Circadian oscillators contribute to the transport of iron ions. Based on the analysis of chlorophyll content, the *cca1 lhy* double mutant and *PRR7* overexpression line are more sensitive to iron excess (600 μM Fe), showing a decrease in chlorophyll content compared to the wild type (Liu et al., 2013). Moreover, the accumulation of photosynthetic protein in *cca1 lhy* was remarkably lower than that of wild-type plants under Fe-deficient conditions (Xu et al., 2019). The root ferric-chelate reductase (FRO) that reduces Fe^3+^ ions to soluble Fe^2+^ ions exhibits a circadian rhythm under constant light, with a peak phase occurring around noon. The *cca1 lhy* mutant exhibits a dramatic decrease in FRO enzyme activity and loss of circadian rhythm. Dex-induced *CCA1* overexpression in *35S:6His-CCA1-GR* transgenic line promotes transcript accumulation of iron homeostasis genes *bHLH038*, *bHLH038*, *bHLH100*, *bHLH101*, *FRO2*, and *IRON-REGULATED TRANSPORTER1* (*IRT1*), and ChIP analysis confirms the binding of CCA1 to the promoter regions of aforementioned genes (Xu et al., 2019). IRT1 is a high-affinity iron transporter responsible for the uptake of iron ions. The *irt1–1* mutant exhibits a lengthened period of circadian rhythm (Hong et al., 2013). The circadian clock regulates *IRT1* rhythmic expression, and the *IRT1* promoter activity reaches its peak in early morning based on *IRT1:LUC* measurement.

TIME FOR COFFEE (TIC) is found in the nucleus and regulates the period length and amplitude of circadian rhythm, possibly by affecting the expression of *LHY* (Ding et al., 2007). The *tic-2* mutant displays a chlorotic phenotype in response to iron status, which can be rescued by exogenous Fe application. TIC inhibits the expression of *FERRITIN 1* (ferritin gene) and iron-responsive genes (Duc et al., 2009). Ferritin mediates oxidative stress response in plants (Briat et al., 2010). Under constant light conditions, *FER1*, *FER3*, and *FER4* transcript accumulation exhibits circadian rhythms, with the peak expression occurring at evening (ZT12), indicating that their transcription was regulated by the circadian clock (Hong et al., 2013; Liu et al., 2013). ChIP-seq analysis in *HA-PRR7*/*prr7–3* material confirms that *FER1*, *FER3* and *FER4* are target genes of PRR7 protein. In summary, the circadian clock gates Fe storage and Fe signaling, thus modulating oxidative stress responses and maintaining daily organelles functions including chloroplast.

In chloroplast, Mg is a key component of chlorophyll and co-factor of many photosynthetic enzymes. Phosphorylation of KaiC protein, a circadian oscillator of cyanobacteria clock, is sensitive to the concentration of Mg (Johnson et al., 2008; Jeong et al., 2019). Low concentration of Mg induces KaiC phosphorylation, and high concentration of Mg leads to dephosphorylation of KaiC (Jeong et al., 2019). In daily photosynthesis, Mg transporter OsMGT3, which is located in chloroplast membrane, assists the Mg intake to maintain the basic functions of chloroplast. In rice chloroplasts, the Mg level oscillates in a 12-h light:12-h dark diurnal cycle with the highest accumulation (peak phase) of diel rhythm occurring in evening (ZT12–16) (Li et al., 2020a). In *osmgt3* mutant, the amplitude of Mg 24-h rhythm is greatly weakened, suggesting that OsMGT3 maintains Mg homeostasis through rhythmic intake of Mg. Moreover, *OsMGT3* transcripts are rhythmically enriched in the leaf blade with the peak of robust diurnal rhythm appearing at dawn. The hypothesis proposed in this study is that OsMGT3 maintain the circadian rhythmic intake of Mg in chloroplast, which contributes to Rubisco activity and photosynthetic carbon assimilation during the daytime (Li et al., 2020a). Recently, it was found that the increase in Mg concentration causes circadian period length to be shortened, indicating that magnesium level is inversely proportional to circadian pace (de Melo et al., 2021). When external supply of Mg is restricted as low as 5 or 25 μM, plant exhibits a very long period length of circadian rhythm and seedlings development is obviously slowed down. In this study, according to the Mg pulse PRC analysis, the 50 μM external pulses do not cause a phase shift of circadian rhythm. However, the studies still reveal that ion-like biochemical oscillators can affect the circadian clock.

## Mechanisms linking the circadian clock and plant defense against pathogens and insect herbivores

During the course of co-evolution with microorganisms and herbivores, plants maintain a temporal resistance to biotic stresses. The circadian clock in Arabidopsis regulates the rhythmic expression of many resistance (*R*)-genes related to innate immunity to resist oomycete pathogen (*Hyaloperonospora arabidopsidis*, *Hpa*) that causes downy mildew (Zhang et al., 2013). When Hpa spores are normally disseminated in nature, *CCA1* plays essential role in the resistance of wild-type Arabidopsis to *Hpa* Emwa1 infection at dawn (Wang et al., 2011). By contrast, *cca1* mutant has more leaves with sporangiophores, regardless of whether they are infected with *Hpa* Emwa1 during the day or night. The *R* gene in Arabidopsis, *RPP4*, exhibits a robust circadian expression with a peak occurring around late night. *Hpa* Emwa1 infection causes arrhythmicity of *RPP4* expression. This study reveals that the plant circadian clock predicts the natural infection time of pathogens and regulates MAMP-triggered basal immunity and *RPP4*-mediated programmed cell death in the hypersensitive responses.

Goodspeed et al. found that the feeding behavior of cabbage loopers (*Trichoplusia ni*) shows robust circadian rhythms under light/dark cycles and continuous dark conditions (Goodspeed et al., 2012). The measurement of consumed diet indicates that feeding started during the subjective day and reaches its peak in the evening, while the amount of diet consumed in early morning is extremely low. From the results, it is speculated that insects avoid feeding from midnight to next morning, and plants are also more defensive against herbivory during the late night and dawn over a 24-h day. During the entrainment that alters light/dark cycle with in-phase or out-of-phase of plants and insects, plants show stronger resistance to herbivory with in-phase than out-of-phase with the entrainment of insects. Overexpression of *CCA1* and knocking out *LUX* causes arrhythmicity in plant and abolished in-phase-dependent enhanced resistance to pests. This result indicates that functional circadian oscillators in plant anticipates the feeding rhythm and prepares the plant to fight against it. The circadian clock regulates the biosynthesis and signal transduction of hormones in response to environmental changes (Atamian and Harmer, 2016). Under free-running conditions, the accumulation of jasmonates (JA) and salicylate (SA) presents diametrically opposite phases in circadian rhythms, JA is enriched during subjective day, and SA is enriched during subjective night. In *aos* and *jar1* mutants lacking active JA accumulation, plants lose their enhanced resistance to *T. ni* loopers during in-phase entrainment, indicating that the rhythmic enrichment of JA during the day is required for plant resistance (Goodspeed et al., 2012). Zhang et al. found that the *lux-1* mutation inhibited the defense against *Pseudomonas syringae* mediated by JA- and SA-signaling (Zhang et al., 2019). Therefore, multiple clock components maintain resistance to biotic stress by regulating hormone signaling.

Further study has found that the circadian clock continues to function in postharvest crops to enhance pest resistance via rhythmic accumulation of antiherbivore metabolite (Goodspeed et al., 2013). The aliphatic glucosinolate accumulation in postharvest cabbage (*Brassica oleracea*) is regulated by the circadian clock with the peak of rhythmic enrichment occurring in subjective day. Compared with constant light or constant darkness conditions, antiherbivore metabolite glucosinolate 4-methylsulfinylbutyl (4MSO) maintains a more robust rhythmicity under the light/dark cycles at 22 °C and 4 °C, but the expression level at 22 °C is higher than that in cold temperature. In addition, the postharvest leaf disk (lettuce and spinach) or the postharvest whole organs (zucchini, sweet potato, carrot, and blueberry) still perceives the different entrainment between in-phase and out-of-phase light/dark cycle, and has enhanced in-phase-dependent resistance to *T. ni* loopers incubation (Goodspeed et al., 2013). In addition, it has recently been discovered that plants resist the rhythmic feeding of green peach aphid (*Myzus persicae*) through the function of *CCA1* alternatively spliced isoforms (Lei et al., 2019). The mechanism hypothesis is that aphid infestation-induced *CCA1* promotes the production of indole glucosinolate. From this analysis, the circadian clock regulates metabolic pathways, including the production of bioactive compounds to resist pests.

In tobacco (*Nicotiana attenuata*) grown under field and glasshouse conditions, the circadian clock contributes to the nitrogen (N) incorporation of three defense compounds caffeoylputrescine (CP), dicaffeoyl spermidine (DCS), and nicotine in response to herbivore *Manduca sext* (Valim et al., 2020). This nicotine response can be inhibited by fatty acid-amino acid conjugates in *Manduca sexta* oral secretions. After knocking out tobacco clock gene *TOC1*, *Manduca sexta* oral secretion-induced JA biosynthesis was obviously reduced, and the expression of defense-related *MYC2*, *COI1*, and *MYB8* gene was also reduced or phase shifted, suggesting that there is a critical role of *TOC1* in relationships between herbivore-induced phenolamide and nicotine in tobacco defense (Li et al., 2018; Valim et al., 2020). Subsequent experiments confirm that mutation of *TOC1* in *Nicotiana attenuata* shifts the N allocation from phenolamides to nicotine (Valim et al., 2020). This study indicates that plants actively allocate N-rich defense components when undergoing pest stress, and fine-tune both vegetative biomass and herbivory defense.

## Concluding remarks

In response to daily and seasonal fluctuating environments, the circadian clock synchronizes many biological processes, such as the opening and closure of stomata, petal & leaf, chloroplast movement, rhythmic growth, tuberization, and photoperiodic flowering (Britz and Briggs, 1976; Holmes and Klien, 1986; Yanovsky et al., 2000; Imaizumi, 2010; Song et al., 2015). Stomata are usually open during the day and closed at night. Stomata conductance and CO_2_ assimilation exhibit rhythmic fluctuations over a 24-h day (Holmes and Klien, 1986; Dodd et al., 2005). This rhythm is maintained even under continuous light conditions, indicating that the circadian clock regulates stomata functions. The circadian amplitude of stomatal movement is known to respond to red and blue light cues in light-dark cycle (Gorton et al., 1993). While maintaining photoperiodic control of seasonal growth and reproduction, stomata is also involved in resistance to abiotic and biotic stresses (Melotto et al., 2008; Qi et al., 2018). Drought causes a decline in photosynthesis and reduces CO_2_ assimilation products, and it is known that drought-induced ABA inhibits the response of stomata to light stimuli under both light-dark cycle and continuous light (Goh et al., 2003). Many known core oscillators of the circadian clock participate extensively in oxidative stress response and disease resistance against *P. syringae* through the regulation of stomatal behavior (Liu et al., 2013; Zhang et al., 2013; Zhang et al., 2019). While there are still many unresolved signal pathways or networks in stomata-mediated stress responses. For example, the circadian clock and photoperiodic signals regulate the circadian rhythm of free calcium in cytoplasm and chloroplast to maintain Ca^2+^ homeostasis (Johnson et al., 1995; Xu et al., 2007). During water deficient stress, calcium ions affect stomatal movement by regulating aquaporin and water flowing and osmotic stress can quickly increase the concentration of cytosolic free calcium (Zhang et al., 2020). These studies do not elucidate how Ca^2+^ homeostasis is maintained in plants grown under stress conditions.

Various environmental stresses weaken and damage plants in agricultural production, therefore breeding resistant crop resources and improving plant adaptability are the keys to ensuring the crop yield. In this review we summarized the critical roles of circadian core oscillators in perception and responses to biotic and abiotic stress signals with information that may be closely relevant to future crop production, such as the longer-term adaptation of agricultural ecosystems, biomass yield, and crop storage after harvest. Using the knowledge of chronobiology to identify germplasm resources and cultivate crop varieties with regionally adaptive circadian clock genotypes and rhythmic phenotypes are the research goals. Moreover, it is known that *CCA1* and *TOC1* regulate the sensitivity of plants to glyphosate, an efficient herbicide (Belbin et al., 2019). Therefore, the effect of herbicide spray timing and dosage on the circadian clock and growth rhythmicity will become one of the concerns in agricultural pest control. It is pointed out in chronoculture theory that the circadian clock outputs are associated with the utilization of water irrigation, fertilizer, and pesticide use in crop production (Steed et al., 2021). Therefore, chronoculture integrates the circadian clock and time-dependent farming and management strategies. In the future, with the help of gene editing technology, the sensitivity of the circadian clock to stress cues and the circadian parameters (phase, amplitude, and period length) will be artificially altered, and it is expected that the novel germplasm resources with optimized circadian clock will improve the adaptability of crops.

## Data Availability

Not applicable.

## Abbreviations

ABA: Abscisic acid
AS: Alternative splicing
CBF1: C-repeat/DRE binding factor 1
CCA1: CIRCADIAN CLOCK ASSOCIATED 1
CHE: CCA1 HIKING EXPEDITION
CO: CONSTANS
COR: Cold-responsive gene
EC: Evening complex
ELF3: EARLY FLOWERING 3
FKF1: FLAVIN BINDING, KELCH REPEAT,F-Box 1
FT: FLOWERING LOCUS T
GI: GIGANTEA
JA: Jasmonate
LHY: LATE ELONGATED HYPOCOTYL
LNK1: NIGHT LIGHT-INDUCIBLE AND CLOCK-REGULATED 1
LUX: LUX ARRHYTHMO
LWD1: LIGHT-REGULATED WD1
MJ: Methyl Jasmonate
N: Nitrogen
phyB: Phytochrome B
PIF: PHYTOCHROME INTERACTING FACTOR
PRC: Phase response curve
PRRs: PSEUDO-RESPONSE REGULATORs
RNAi: RNA interference
RVE4: REVEILLE 4
SA: Salicylate
SAG: Senescence-associated gene
TIC: TIME FOR COFFEE
TOC1: TIMING OF CAB EXPRESSION 1
TTFLs: Transcription-translation feedback loops
ZTL: ZEITLUPE

## References

[CR1] Adams S, Grundy J, Veflingstad SR, Dyer NP, Hannah MA, Ott S, Carre IA (2018). Circadian control of abscisic acid biosynthesis and signalling pathways revealed by genome-wide analysis of LHY binding targets. New Phytol.

[CR2] Andres-Colas N, Perea-Garcia A, Puig S, Penarrubia L (2010). Deregulated copper transport affects Arabidopsis development especially in the absence of environmental cycles. Plant Physiol.

[CR3] Atamian HS, Harmer SL (2016). Circadian regulation of hormone signaling and plant physiology. Plant Mol Biol.

[CR4] Baek D, Kim WY, Cha JY, Park HJ, Shin G, Park J, Lim CJ, Chun HJ, Li N, Kim DH, Lee SY, Pardo JM, Kim MC, Yun DJ (2020). The GIGANTEA-ENHANCED EM LEVEL complex enhances drought tolerance via regulation of abscisic acid synthesis. Plant Physiol.

[CR5] Belbin FE, Hall GJ, Jackson AB, Schanschieff FE, Archibald G, Formstone C, Dodd AN (2019). Plant circadian rhythms regulate the effectiveness of a glyphosate-based herbicide. Nat Commun.

[CR6] Bieniawska Z, Espinoza C, Schlereth A, Sulpice R, Hincha DK, Hannah MA (2008). Disruption of the Arabidopsis circadian clock is responsible for extensive variation in the cold-responsive transcriptome. Plant Physiol.

[CR7] Boden SA, Weiss D, Ross JJ, Davies NW, Trevaskis B, Chandler PM, Swain SM (2014). EARLY FLOWERING3 regulates FLOWERING in spring barley by mediating gibberellin production and FLOWERING LOCUS T expression. Plant Cell.

[CR8] Bonnot T, Blair EJ, Cordingley SJ, Nagel DH (2021). Circadian coordination of cellular processes and abiotic stress responses. Curr Opin Plant Biol.

[CR9] Box MS, Huang BE, Domijan M, Jaeger KE, Khattak AK, Yoo SJ, Sedivy EL, Jones DM, Hearn TJ, Webb AA, Grant A, Locke JC, Wigge PA (2015). ELF3 controls thermoresponsive growth in Arabidopsis. Curr Biol.

[CR10] Briat JF, Duc C, Ravet K, Gaymard F (2010). Ferritins and iron storage in plants. Biochim Biophys Acta.

[CR11] Britz SJ, Briggs WR (1976). Circadian rhythms of chloroplast orientation and photosynthetic capacity in ulva. Plant Physiol.

[CR12] Bruce VG, Weight F, Pittendrigh CS (1960). Resetting the sporulation rhythm in Pilobolus with short light flashes of high intensity. Science.

[CR13] Cao S, Ye M, Jiang S (2005). Involvement of *GIGANTEA* gene in the regulation of the cold stress response in Arabidopsis. Plant Cell Rep.

[CR14] Carré IA (2002). ELF3: a circadian safeguard to buffer effects of light. Trends Plant Sci.

[CR15] Cha JY, Kim J, Kim TS, Zeng Q, Wang L, Lee SY, Kim WY, Somers DE (2017). GIGANTEA is a co-chaperone which facilitates maturation of ZEITLUPE in the Arabidopsis circadian clock. Nat Commun.

[CR16] Chen WW, Takahashi N, Hirata Y, Ronald J, Porco S, Davis SJ, Nusinow DA, Kay SA, Mas P (2020). A mobile ELF4 delivers circadian temperature information from shoots to roots. Nat Plants.

[CR17] Chen YY, Wang Y, Shin LJ, Wu JF, Shanmugam V, Tsednee M, Lo JC, Chen CC, Wu SH, Yeh KC (2013). Iron is involved in the maintenance of circadian period length in Arabidopsis. Plant Physiol.

[CR18] Cheng Q, Gan Z, Wang Y, Lu S, Hou Z, Li H, Xiang H, Liu B, Kong F, Dong L (2020). The soybean gene J contributes to salt stress tolerance by up-regulating salt-responsive genes. Front Plant Sci.

[CR19] Covington MF, Maloof JN, Straume M, Kay SA, Harmer SL (2008). Global transcriptome analysis reveals circadian regulation of key pathways in plant growth and development. Genome Biol.

[CR20] Covington MF, Panda S, Liu XL, Strayer CA, Wagner DR, Kay SA (2001). ELF3 modulates resetting of the circadian clock in *Arabidopsis*. Plant Cell.

[CR21] Creux N, Harmer S (2019). Circadian rhythms in plants. Cold Spring Harb Perspect Biol.

[CR22] de Melo JRF, Gutsch A, Caluwe T, Leloup JC, Gonze D, Hermans C, Webb AAR, Verbruggen N (2021). Magnesium maintains the length of the circadian period in Arabidopsis. Plant Physiol.

[CR23] Desai JS, Lawas LMF, Valente AM, Leman AR, Grinevich DO, Jagadish SVK, Doherty CJ (2021). Warm nights disrupt transcriptome rhythms in field-grown rice panicles. Proc Natl Acad Sci U S A.

[CR24] Ding L, Wang S, Song ZT, Jiang Y, Han JJ, Lu SJ, Li L, Liu JX (2018). Two B-Box domain proteins, BBX18 and BBX23, interact with ELF3 and regulate Thermomorphogenesis in Arabidopsis. Cell Rep.

[CR25] Ding Z, Millar AJ, Davis AM, Davis SJ (2007). TIME FOR COFFEE encodes a nuclear regulator in the *Arabidopsis thaliana* circadian clock. Plant Cell.

[CR26] Dodd AN, Salathia N, Hall A, Kevei E, Toth R, Nagy F, Hibberd JM, Millar AJ, Webb AAR (2005). Plant circadian clocks increase photosynthesis, growth, survival, and competitive advantage. Science.

[CR27] Dong MA, Farré EM, Thomashow MF (2011). CIRCADIAN CLOCK-ASSOCIATED 1 and LATE ELONGATED HYPOCOTYL regulate expression of the C-REPEAT BINDING FACTOR (CBF) pathway in Arabidopsis. Proc Natl Acad Sci U S A.

[CR28] Duc C, Cellier F, Lobreaux S, Briat JF, Gaymard F (2009). Regulation of iron homeostasis in Arabidopsis thaliana by the clock regulator time for coffee. J Biol Chem.

[CR29] Edwards KD, Lynn JR, Gyula P, Nagy F, Millar AJ (2005). Natural allelic variation in the temperature-compensation mechanisms of the Arabidopsis thaliana circadian clock. Genetics.

[CR30] Endo M, Shimizu H, Nohales MA, Araki T, Kay SA (2014). Tissue-specific clocks in Arabidopsis show asymmetric coupling. Nature.

[CR31] Ezer D, Jung JH, Lan H, Biswas S, Gregoire L, Box MS, Charoensawan V, Cortijo S, Lai X, Stockle D, Zubieta C, Jaeger KE, Wigge PA (2017). The evening complex coordinates environmental and endogenous signals in Arabidopsis. Nat Plants.

[CR32] Feeney KA, Hansen LL, Putker M, Olivares-Yanez C, Day J, Eades LJ, Larrondo LF, Hoyle NP, O'Neill JS, van Ooijen G (2016). Daily magnesium fluxes regulate cellular timekeeping and energy balance. Nature.

[CR33] Filichkin SA, Mockler TC (2012). Unproductive alternative splicing and nonsense mRNAs: a widespread phenomenon among plant circadian clock genes. Biol Direct.

[CR34] Fornara F, de Montaigu A, Sanchez-Villarreal A, Takahashi Y, Ver Loren van Themaat E, Huettel B, Davis SJ, Coupland G (2015). The GI-CDF module of Arabidopsis affects freezing tolerance and growth as well as flowering. Plant J.

[CR35] Franklin KA, Lee SH, Patel D, Kumar SV, Spartz AK, Gu C, Ye S, Yu P, Breen G, Cohen JD, Wigge PA, Gray WM (2011). PHYTOCHROME-INTERACTING FACTOR 4 (PIF4) regulates auxin biosynthesis at high temperature. Proc Natl Acad Sci U S A.

[CR36] Fujiwara S, Wang L, Han L, Suh SS, Salomé PA, McClung CR, Somers DE (2008). Post-translational regulation of the circadian clock through selective proteolysis and phosphorylation of pseudo-response regulator proteins. J Biol Chem.

[CR37] Gendron JM, Pruneda-Paz JL, Doherty CJ, Gross AM, Kang SE, Kay SA (2012). Arabidopsis circadian clock protein, TOC1, is a DNA-binding transcription factor. Proc Natl Acad Sci U S A.

[CR38] Gil KE, Kim WY, Lee HJ, Faisal M, Saquib Q, Alatar AA, Park CM (2017). ZEITLUPE contributes to a Thermoresponsive protein quality control system in Arabidopsis. Plant Cell.

[CR39] Goh CH, Nam HG, Park YS (2003). Stress memory in plants: a negative regulation of stomatal response and transient induction of rd22 gene to light in abscisic acid-entrained Arabidopsis plants. Plant J.

[CR40] Gol L, Haraldsson EB, von Korff M (2021). Ppd-H1 integrates drought stress signals to control spike development and flowering time in barley. J Exp Bot.

[CR41] Goodspeed D, Chehab EW, Min-Venditti A, Braam J, Covington MF (2012). Arabidopsis synchronizes jasmonate-mediated defense with insect circadian behavior. Proc Natl Acad Sci U S A.

[CR42] Goodspeed D, Liu JD, Chehab EW, Sheng Z, Francisco M, Kliebenstein DJ, Braam J (2013). Postharvest circadian entrainment enhances crop pest resistance and phytochemical cycling. Curr Biol.

[CR43] Gorton HL, Williams WE, Assmann SM (1993). Circadian rhythms in stomatal responsiveness to red and blue light. Plant Physiol.

[CR44] Gould PD, Domijan M, Greenwood M, Tokuda IT, Rees H, Kozma-Bognar L, Hall AJ, Locke JC (2018). Coordination of robust single cell rhythms in the Arabidopsis circadian clock via spatial waves of gene expression. Elife.

[CR45] Gray WM, Östin A, Sandberg G, Romano CP, Estelle M (1998). High temperature promotes auxin-mediated hypocotyl elongation in *Arabidopsis*. Proc Natl Acad Sci U S A.

[CR46] Greenham K, McClung CR (2015). Integrating circadian dynamics with physiological processes in plants. Nat Rev Genet.

[CR47] Grundy J, Stoker C, Carre IA (2015). Circadian regulation of abiotic stress tolerance in plants. Front Plant Sci.

[CR48] Gutierrez RA, Stokes TL, Thum K, Xu X, Obertello M, Katari MS, Tanurdzic M, Dean A, Nero DC, McClung CR, Coruzzi GM (2008). Systems approach identifies an organic nitrogen-responsive gene network that is regulated by the master clock control gene CCA1. Proc Natl Acad Sci U S A.

[CR49] Hanano S, Domagalska MA, Nagy F, Davis SJ (2006). Multiple phytohormones influence distinct parameters of the plant circadian clock. Genes Cells.

[CR50] Harmer SL, Hogenesch JB, Straume M, Chang HS, Han B, Zhu T, Wang X, Kreps JA, Kay SA (2000). Orchestrated transcription of key pathways in Arabidopsis by the circadian clock. Science.

[CR51] Haydon MJ, Mielczarek O, Robertson FC, Hubbard KE, Webb AA (2013). Photosynthetic entrainment of the Arabidopsis thaliana circadian clock. Nature.

[CR52] Hermans C, Vuylsteke M, Coppens F, Craciun A, Inze D, Verbruggen N (2010). Early transcriptomic changes induced by magnesium deficiency in Arabidopsis thaliana reveal the alteration of circadian clock gene expression in roots and the triggering of abscisic acid-responsive genes. New Phytol.

[CR53] Holmes MG, Klien WH (1986). Photocontrol of dark circadian rhythms in stomata of *Phaseolus vulgaris* L. Plant Physiol.

[CR54] Hong S, Kim SA, Guerinot ML, McClung CR (2013). Reciprocal interaction of the circadian clock with the iron homeostasis network in Arabidopsis. Plant Physiol.

[CR55] Hsu PY, Harmer SL (2014). Wheels within wheels: the plant circadian system. Trends Plant Sci.

[CR56] Huang H, Alvarez S, Bindbeutel R, Shen Z, Naldrett MJ, Evans BS, Briggs SP, Hicks LM, Kay SA, Nusinow DA (2016). Identification of evening complex associated proteins in Arabidopsis by affinity purification and mass spectrometry. Mol Cell Proteomics.

[CR57] Huang W, Pérez-García P, Pokhilko A, Millar AJ, Antoshechkin I, Riechmann JL, Mas P (2012). Mapping the core of the Arabidopsis circadian clock defines the network structure of the oscillator. Science.

[CR58] Ibanez C, Kozarewa I, Johansson M, Ogren E, Rohde A, Eriksson ME (2010). Circadian clock components regulate entry and affect exit of seasonal dormancy as well as winter hardiness in Populus trees. Plant Physiol.

[CR59] Imaizumi T (2010). Arabidopsis circadian clock and photoperiodism: time to think about location. Curr Opin Plant Biol.

[CR60] Imaizumi T, Schultz TF, Harmon FG, Ho LA, Kay SA (2005). FKF1 F-box protein mediates cyclic degradation of a repressor of CONSTANS in Arabidopsis. Science.

[CR61] Imaizumi T, Tran HG, Swartz TE, Briggs WR, Kay SA (2003). FKF1 is essential for photoperiodic-specific light signalling in Arabidopsis. Nature.

[CR62] James AB, Syed NH, Bordage S, Marshall J, Nimmo GA, Jenkins GI, Herzy P, Brown JWS, Nimmo HG (2012). Alternative splicing mediates responses of the *Arabidopsis* circadian clock to temperature changes. Plant Cell.

[CR63] Jeong YM, Dias C, Diekman C, Brochon H, Kim P, Kaur M, Kim YS, Jang HI, Kim YI (2019). Magnesium regulates the circadian oscillator in Cyanobacteria. J Biol Rhythm.

[CR64] Jiang BC, Shi YT, Zhang XY, Xin XY, Qi LJ, Guo HW, Li JG, Yang SH (2017). PIF3 is a negative regulator of the CBF pathway and freezing tolerance in Arabidopsis. Proc Natl Acad Sci U S A.

[CR65] Jiang Y, Yang C, Huang S, Xie F, Xu Y, Liu C, Li L (2019). The ELF3-PIF7 interaction mediates the circadian gating of the shade response in Arabidopsis. iScience.

[CR66] Johnson CH (1999). Forty years of PRCs--what have we learned?. Chronobiol Intl.

[CR67] Johnson CH, Knight MR, Kondo T, Masson P, Sedbrook J, Haley A, Trewavas A (1995). Circadian oscillations of cytosolic and chloroplastic free calcium in plants. Science.

[CR68] Johnson CH, Mori T, Xu Y (2008). A cyanobacterial circadian clockwork. Curr Biol.

[CR69] Jung JH, Barbosa AD, Hutin S, Kumita JR, Gao MJ, Derwort D, Silva CS, Lai XL, Pierre E, Geng F, Kim SB, Baek S, Zubieta C, Jaeger KE, Wigge PA (2020). A prion-like domain in ELF3 functions as a thermosensor inArabidopsis. Nature.

[CR70] Jung JH, Domijan M, Klose C, Biswas S, Ezer D, Gao M, Khattak AK, Box MS, Charoensawan V, Cortijo S, Kumar M, Grant A, Locke JC, Schafer E, Jaeger KE, Wigge PA (2016). Phytochromes function as thermosensors in Arabidopsis. Science.

[CR71] Kamioka M, Takao S, Suzuki T, Taki K, Higashiyama T, Kinoshita T, Nakamichi N (2016). Direct repression of evening genes by CIRCADIAN CLOCK-ASSOCIATED1 in the Arabidopsis circadian clock. Plant Cell.

[CR72] Kidokoro S, Hayashi K, Haraguchi H, Ishikawa T, Soma F, Konoura I, Toda S, Mizoi J, Suzuki T, Shinozaki K, Yamaguchi-Shinozaki K (2021). Posttranslational regulation of multiple clock-related transcription factors triggers cold-inducible gene expression in Arabidopsis. Proc Natl Acad Sci U S A.

[CR73] Kim TS, Wang L, Kim YJ, Somers DE (2020). Compensatory mutations in GI and ZTL may modulate temperature compensation in the circadian clock. Plant Physiol.

[CR74] Kim W-Y, Ali Z, Park HJ, Park SJ, Cha J-Y, Perez-Hormaeche J, Quintero FJ, Shin G, Kim MR, Qiang Z, Ning L, Park HC, Lee SY, Bressan RA, Pardo JM, Bohnert HJ, Yun D-J (2013). Release of SOS2 kinase from sequestration with GIGANTEA determines salt tolerance in *Arabidopsis*. Nat Commun.

[CR75] Kim W-Y, Fujiwara S, Suh S-S, Kim J, Kim Y, Han L, David K, Putterill J, Nam HG, Somers DE (2007). ZEITLUPE is a circadian photoreceptor stabilized by GIGANTEA in blue light. Nature.

[CR76] Koini MA, Alvey L, Allen T, Tilley CA, Harberd NP, Whitelam GC, Franklin KA (2009). High temperature-mediated adaptations in plant architecture require the bHLH transcription factor PIF4. Curr Biol.

[CR77] Lau OS, Huang X, Charron J-B, Lee J-H, Li G, Deng XW (2011). Interaction of *Arabidopsis* DET1 with CCA1 and LHY in mediating transcriptional repression in the plant circadian clock. Mol Cell.

[CR78] Lee C-M, Thomashow MF (2012). Photoperiodic regulation of the C-repeat binding factor (CBF) cold acclimation pathway and freezing tolerance in *Arabidopsis thaliana*. Proc Natl Acad Sci U S A.

[CR79] Lee KH, Piao HL, Kim HY, Choi SM, Jiang F, Hartung W, Hwang I, Kwak JM, Lee IJ (2006). Activation of glucosidase via stress-induced polymerization rapidly increases active pools of abscisic acid. Cell.

[CR80] Legnaioli T, Cuevas J, Mas P (2009). TOC1 functions as a molecular switch connecting the circadian clock with plant responses to drought. EMBO J.

[CR81] Legris M, Klose C, Burgie ES, Rojas CC, Neme M, Hiltbrunner A, Wigge PA, Schafer E, Vierstra RD, Casal JJ (2016). Phytochrome B integrates light and temperature signals in Arabidopsis. Science.

[CR82] Lei J, Jayaprakasha GK, Singh J, Uckoo R, Borrego EJ, Finlayson S, Kolomiets M, Patil BS, Braam J, Zhu-Salzman K (2019). CIRCADIAN CLOCK-ASSOCIATED1 controls resistance to aphids by altering indole Glucosinolate production. Plant Physiol.

[CR83] Li BJ, Gao ZH, Liu XY, Sun DY, Tang WQ (2019). Transcriptional profiling reveals a time-of-Day-specific role of REVEILLE 4/8 in regulating the first wave of heat shock-induced gene expression in Arabidopsis. Plant Cell.

[CR84] Li J, Yokosho K, Liu S, Cao HR, Yamaji N, Zhu XG, Liao H, Ma JF, Chen ZC (2020). Diel magnesium fluctuations in chloroplasts contribute to photosynthesis in rice. Nat Plants.

[CR85] Li R, Llorca LC, Schuman MC, Wang Y, Wang L, Joo Y, Wang M, Vassao DG, Baldwin IT (2018). ZEITLUPE in the roots of wild tobacco regulates Jasmonate-mediated nicotine biosynthesis and resistance to a generalist herbivore. Plant Physiol.

[CR86] Li Y, Wang L, Yuan L, Song Y, Sun J, Jia Q, Xie Q, Xu X (2020). Molecular investigation of organ-autonomous expression of Arabidopsis circadian oscillators. Plant Cell Environ.

[CR87] Liu T, Carlsson J, Takeuchi T, Newton L, Farré EM (2013). Direct regulation of abiotic responses by the *Arabidopsis* circadian clock component PRR7. Plant J.

[CR88] Liu TL, Newton L, Liu MJ, Shiu SH, Farre EM (2016). A G-Box-like motif is necessary for transcriptional regulation by circadian Pseudo-response regulators in Arabidopsis. Plant Physiol.

[CR89] Love J, Dodd AN, Webb AAR (2004). Circadian and diurnal calcium oscillations encode photoperiodic information in Arabidopsis. Plant Cell.

[CR90] Lu S, Zhao X, Hu Y, Liu S, Nan H, Li X, Fang C, Cao D, Shi X, Kong L, Su T, Zhang F, Li S, Wang Z, Yuan X, Cober ER, Weller JL, Liu B, Hou X, Tian Z, Kong F (2017). Natural variation at the soybean J locus improves adaptation to the tropics and enhances yield. Nat Genet.

[CR91] Ma Y, Gil S, Grasser KD, Mas P (2018). Targeted recruitment of the basal transcriptional machinery by LNK clock components controls the circadian rhythms of nascent RNAs in Arabidopsis. Plant Cell.

[CR92] Markham KK, Greenham K (2021). Abiotic stress through time. New Phytol.

[CR93] Martin G, Rovira A, Veciana N, Soy J, Toledo-Ortiz G, Gommers CMM, Boix M, Henriques R, Minguet EG, Alabadi D, Halliday KJ, Leivar P, Monte E (2018). Circadian waves of transcriptional repression shape PIF-regulated photoperiod-responsive growth in Arabidopsis. Curr Biol.

[CR94] McClung CR (2019). The plant circadian oscillator. Biology (Basel).

[CR95] McWatters HG, Bastow RM, Hall A, Millar AJ (2000). The *ELF3 zeitnehmer* regulates light signalling to the circadian clock. Nature.

[CR96] Melotto M, Underwood W, He SY (2008). Role of stomata in plant innate immunity and foliar bacterial diseases. Annu Rev Phytopathol.

[CR97] Millar AJ, Carré IA, Strayer CA, Chua N-H, Kay SA (1995). Circadian clock mutants in *Arabidopsis* identified by luciferase imaging. Science.

[CR98] Mizuno T, Takeuchi A, Nomoto Y, Nakamichi N, Yamashino T (2014). The *LNK1* night light-inducible and clock-regulated gene is induced also in response to warm-night through the circadian clock nighttime repressor in *Arabidopsis thaliana*. Plant Signal Behav.

[CR99] Mizuno T, Yamashino T (2008). Comparative transcriptome of diurnally oscillating genes and hormone-responsive genes in *Arabidopsis thaliana*: insight into circadian clock-controlled daily responses to common ambient stresses in plants. Plant Cell Physiol..

[CR100] Mockler TC, Michael TP, Priest HD, Shen R, Sullivan CM, Givan SA, McEntee C, Kay SA, Chory J (2007). The diurnal project: diurnal and circadian expression profiling, model-based pattern matching, and promoter analysis. Cold Spring Harb Symp Quant Biol.

[CR101] Nagel DH, Doherty CJ, Pruneda-Paz JL, Schmitz RJ, Ecker JR, Kay SA (2015). Genome-wide identification of CCA1 targets uncovers an expanded clock network in Arabidopsis. Proc Natl Acad Sci U S A.

[CR102] Nakamichi N, Kiba T, Henriques R, Mizuno T, Chua N-H, Sakakibara H (2010). PSEUDO-RESPONSE REGULATORS 9, 7 and 5 are transcriptional repressors in the *Arabidopsis* circadian clock. Plant Cell.

[CR103] Nakamichi N, Kita M, Ito S, Sato E, Yamashino T, Mizuno T (2005). PSEUDO-RESPONSE REGULATORS, PRR9, PRR7 and PRR5, together play essential roles close to the circadian clock of *Arabidopsis thaliana*. Plant Cell Physiol..

[CR104] Nakamichi N, Kusano M, Fukushima A, Kita M, Ito S, Yamashino T, Saito K, Sakakibara H, Mizuno T (2009). Transcript profiling of an Arabidopsis PSEUDO RESPONSE REGULATOR arrhythmic triple mutant reveals a role for the circadian clock in cold stress response. Plant Cell Physiol.

[CR105] Nelson DC, Lasswell J, Rogg LE, Cohen MA, Bartel B (2000). FKF1, a clock-controlled gene that regulates the transition to flowering in *Arabidopsis*. Cell.

[CR106] Ni Z, Kim E-D, Ha M, Lackey E, Liu J, Zhang Y, Sun Q, Chen ZJ (2009). Altered circadian rhythms regulate growth vigour in hybrids and allopolyploids. Nature.

[CR107] Nohales MA, Kay SA (2016). Molecular mechanisms at the core of the plant circadian oscillator. Nat Struct Mol Biol.

[CR108] Nusinow DA, Helfer A, Hamilton EE, King JJ, Imaizumi T, Schultz TF, Farre EM, Kay SA (2011). The ELF4-ELF3-LUX complex links the circadian clock to diurnal control of hypocotyl growth. Nature.

[CR109] Para A, Farré EM, Imaizumi T, Pruneda-Paz JL, Harmon FG, Kay SA (2007). PRR3 is a vascular regulator of TOC1 stability in the *Arabidopsis* circadian clock. Plant Cell.

[CR110] Park HJ, Qiang Z, Kim WY, Yun DJ (2016). Diurnal and circadian regulation of salt tolerance in Arabidopsis. J Plant Biol.

[CR111] Pittendrigh CS (1954). On the temperature independence in the clock system controlling emergence time in *Drosophila*. Proc Natl Acad Sci U S A.

[CR112] Pittendrigh CS (1960). Circadian rhythms and the circadian organization of living systems. Cold Spring Harb Symp Quant Biol.

[CR113] Pruneda-Paz JL, Breton G, Para A, Kay SA (2009). A functional genomics approach reveals CHE as a novel component of the Arabidopsis circadian clock. Science.

[CR114] Pudasaini A, Shim JS, Song YH, Shi H, Kiba T, Somers DE, Imaizumi T, Zoltowski BD (2017). Kinetics of the LOV domain of ZEITLUPE determine its circadian function in Arabidopsis. Elife.

[CR115] Qi J, Song CP, Wang B, Zhou J, Kangasjarvi J, Zhu JK, Gong Z (2018). Reactive oxygen species signaling and stomatal movement in plant responses to drought stress and pathogen attack. J Integr Plant Biol.

[CR116] Qiu Y, Pasoreck EK, Yoo CY, He J, Wang H, Bajracharya A, Li M, Larsen HD, Cheung S, Chen M (2021). RCB initiates Arabidopsis thermomorphogenesis by stabilizing the thermoregulator PIF4 in the daytime. Nat Commun.

[CR117] Sai J, Johnson CH (1999). Different circadian oscillators control ca(2+) fluxes and *Lhcb* gene expression. Proc Natl Acad Sci U S A.

[CR118] Sakuraba Y, Bulbul S, Piao W, Choi G, Paek NC (2017). Arabidopsis EARLY FLOWERING3 increases salt tolerance by suppressing salt stress response pathways. Plant J.

[CR119] Sakuraba Y, Han SH, Yang HJ, Piao W, Paek NC (2016). Mutation of Rice early Flowering3.1 (OsELF3.1) delays leaf senescence in rice. Plant Mol Biol.

[CR120] Salomé PA, McClung CR (2005). What makes the Arabidopsis clock tick on time?: a review on entrainment. Plant Cell Environ.

[CR121] Salomé PA, Oliva M, Weigel D, Krämer U (2013). Circadian clock adjustment to plant iron status depends on chloroplast and phytochrome function. EMBO J.

[CR122] Salomé PA, Weigel D, McClung CR (2010). The role of the *Arabidopsis* morning loop components CCA1, LHY, PRR7 and PRR9 in temperature compensation. Plant Cell.

[CR123] Sanchez SE, Petrillo E, Beckwith EJ, Zhang X, Rugnone ML, Hernando CE, Cuevas JC, Godoy Herz MA, Depetris-Chauvin A, Simpson CG, Brown JWS, Cerdán PD, Borevitz JO, Mas P, Ceriani MF, Kornblihtt AR, Yanovsky MJ (2010). A methyl transferase links the circadian clock to the regulation of alternative splicing. Nature.

[CR124] Sanchez SE, Rugnone ML, Kay SA (2020). Light perception: a matter of time. Mol Plant.

[CR125] Schultz TF, Kiyosue T, Yanovsky M, Wada M, Kay SA (2001). A role for LKP2 in the circadian clock of Arabidopsis. Plant Cell.

[CR126] Seo PJ, Mas P (2014). Multiple layers of posttranslational regulation refine circadian clock activity in Arabidopsis. Plant Cell.

[CR127] Seo PJ, Mas P (2015). STRESSing the role of the plant circadian clock. Trends Plant Sci.

[CR128] Seo PJ, Park MJ, Lim MH, Kim SG, Lee M, Baldwin IT, Park CM (2012). A self-regulatory circuit of CIRCADIAN CLOCK-ASSOCIATED1 underlies the circadian clock regulation of temperature responses in Arabidopsis. Plant Cell.

[CR129] Somers DE, Devlin P, Kay SA (1998). Phytochromes and cryptochromes in the entrainment of the *Arabidopsis* circadian clock. Science.

[CR130] Somers DE, Schultz TF, Milnamow M, Kay SA (2000). ZEITLUPE encodes a novel clock-associated PAS protein from Arabidopsis. Cell.

[CR131] Song YH, Shim JS, Kinmonth-Schultz HA, Imaizumi T (2015). Photoperiodic flowering: time measurement mechanisms in leaves. Annu Rev Plant Biol.

[CR132] Soy J, Leivar P, Gonzalez-Schain N, Martin G, Diaz C, Sentandreu M, Al-Sady B, Quail PH, Monte E (2016). Molecular convergence of clock and photosensory pathways through PIF3-TOC1 interaction and co-occupancy of target promoters. Proc Natl Acad Sci U S A.

[CR133] Steed G, Ramirez DC, Hannah MA, Webb AAR (2021). Chronoculture, harnessing the circadian clock to improve crop yield and sustainability. Science.

[CR134] Sun Q, Wang S, Xu G, Kang X, Zhang M, Ni M (2019). SHB1 and CCA1 interaction desensitizes light responses and enhances thermomorphogenesis. Nat Commun.

[CR135] Takahashi N, Hirata Y, Aihara K, Mas P (2015). A hierarchical multi-oscillator network orchestrates the Arabidopsis circadian system. Cell.

[CR136] Thain SC, Hall A, Millar AJ (2000). Functional independence of circadian clocks that regulate plant gene expression. Curr Biol.

[CR137] Thines B, Harmon FG (2010). Ambient temperature response establishes ELF3 as a required component of the core Arabidopsis circadian clock. Proc Natl Acad Sci U S A.

[CR138] Valim H, Dalton H, Joo Y, McGale E, Halitschke R, Gaquerel E, Baldwin IT, Schuman MC (2020). TOC1 in Nicotiana attenuata regulates efficient allocation of nitrogen to defense metabolites under herbivory stress. New Phytol.

[CR139] Valim HF, McGale E, Yon F, Halitschke R, Fragoso V, Schuman MC, Baldwin IT (2019). The clock gene TOC1 in shoots, not roots, determines fitness of Nicotiana attenuata under drought. Plant Physiol.

[CR140] Wang K, Bu T, Cheng Q, Dong L, Su T, Chen Z, Kong F, Gong Z, Liu B, Li M (2021). Two homologous LHY pairs negatively control soybean drought tolerance by repressing the abscisic acid responses. New Phytol.

[CR141] Wang L, Fujiwara S, Somers DE (2010). PRR5 regulates phosphorylation, nuclear import and subnuclear localization of TOC1 in the Arabidopsis circadian clock. EMBO J.

[CR142] Wang L, Kim J, Somers DE (2013). Transcriptional corepressor TOPLESS complexes with pseudoresponse regulator proteins and histone deacetylases to regulate circadian transcription. Proc Natl Acad Sci U S A.

[CR143] Wang P, Cui X, Zhao C, Shi L, Zhang G, Sun F, Cao X, Yuan L, Xie Q, Xu X (2017). COR27 and COR28 encode nighttime repressors integrating Arabidopsis circadian clock and cold response. J Integr Plant Biol.

[CR144] Wang W, Barnaby JY, Tada Y, Li H, Tör M, Caldelari D, Lee D-U, Fu X-D, Dong X (2011). Timing of plant immune responses by a central circadian regulator. Nature.

[CR145] Wang Y, He Y, Su C, Zentella R, Sun TP, Wang L (2020). Nuclear localized O-Fucosyltransferase SPY facilitates PRR5 proteolysis to fine-tune the pace of Arabidopsis circadian clock. Mol Plant.

[CR146] Wang Y, Yuan L, Su T, Wang Q, Gao Y, Zhang S, Jia Q, Yu G, Fu Y, Cheng Q, Liu B, Kong F, Zhang X, Song CP, Xu X, Xie Q (2020). Light- and temperature-entrainable circadian clock in soybean development. Plant Cell Environ.

[CR147] Webb AAR, Seki M, Satake A, Caldana C (2019). Continuous dynamic adjustment of the plant circadian oscillator. Nat Commun.

[CR148] Wei H, Wang X, He Y, Xu H, Wang L (2021). Clock component OsPRR73 positively regulates rice salt tolerance by modulating OsHKT2;1-mediated sodium homeostasis. EMBO J.

[CR149] Wu JF, Tsai HL, Joanito I, Wu YC, Chang CW, Li YH, Wang Y, Hong JC, Chu JW, Hsu CP, Wu SH (2016). LWD-TCP complex activates the morning gene CCA1 in Arabidopsis. Nat Commun.

[CR150] Wu J-F, Wang Y, Wu S-H (2008). Two new clock proteins, LWD1 and LWD2, regulate Arabidopsis photoperiodic flowering. Plant Physiol.

[CR151] Xie Q, Lou P, Hermand V, Aman R, Park HJ, Yun DJ, Kim WY, Salmela MJ, Ewers BE, Weinig C, Khan SL, Schaible DL, McClung CR (2015). Allelic polymorphism of GIGANTEA is responsible for naturally occurring variation in circadian period in Brassica rapa. Proc Natl Acad Sci U S A.

[CR152] Xie Q, Wang P, Liu X, Yuan L, Wang L, Zhang C, Li Y, Xing H, Zhi L, Yue Z, Zhao C, McClung CR, Xu X (2014). LNK1 and LNK2 are transcriptional coactivators in the Arabidopsis circadian oscillator. Plant Cell.

[CR153] Xu G, Jiang Z, Wang H, Lin R (2019). The central circadian clock proteins CCA1 and LHY regulate iron homeostasis in Arabidopsis. J Integr Plant Biol.

[CR154] Xu X, Hotta CT, Dodd AN, Love J, Sharrock R, Lee YW, Xie Q, Johnson CH, Webb AA (2007). Distinct light and clock modulation of cytosolic free Ca2+ oscillations and rhythmic CHLOROPHYLL a/B BINDING PROTEIN2 promoter activity in Arabidopsis. Plant Cell.

[CR155] Xu X, Xie Q, McClung CR (2010). Robust circadian rhythms of gene expression in Brassica rapa tissue culture. Plant Physiol.

[CR156] Xu X, Yuan L, Xie Q (2022). Circadian rhythm: phase response curve and light entrainment. Methods Mol Biol.

[CR157] Xu X, Yuan L, Yang X, Zhang X, Wang L, Xie Q (2022b) Circadian clock in plants: linking timing to fitness. J Integr Plant Biol. 10.1111/jipb.13230 10.1111/jipb.13230 35088570

[CR158] Yan J, Li S, Kim YJ, Zeng Q, Radziejwoski A, Wang L, Nomura Y, Nakagami H, Somers DE (2021). TOC1 clock protein phosphorylation controls complex formation with NF-YB/C to repress hypocotyl growth. EMBO J.

[CR159] Yang M, Han X, Yang J, Jiang Y, Hu Y (2021). The Arabidopsis circadian clock protein PRR5 interacts with and stimulates ABI5 to modulate abscisic acid signaling during seed germination. Plant Cell.

[CR160] Yang Y, Guo Y (2018). Unraveling salt stress signaling in plants. J Integr Plant Biol.

[CR161] Yanovsky MJ, Izaguirre M, Wagmaister JA, Gatz C, Jackson SD, Thomas B, Casal JJ (2000). Phytochrome a resets the circadian clock and delays tuber formation under long days in potato. Plant J.

[CR162] Yuan L, Hu Y, Li S, Xie Q, Xu X (2020). PRR9 and PRR7 negatively regulate the expression of EC components under warm temperature in roots. Plant Signal Behav.

[CR163] Yuan L, Xie GZ, Zhang S, Li B, Wang X, Li Y, Liu T, Xu X (2021). GmLCLs negatively regulate ABA perception and signalling genes in soybean leaf dehydration response. Plant Cell Environ.

[CR164] Yuan L, Yu Y, Liu M, Song Y, Li H, Sun J, Wang Q, Xie Q, Wang L, Xu X (2021). BBX19 fine-tunes the circadian rhythm by interacting with PSEUDO-RESPONSE REGULATOR proteins to facilitate their repressive effect on morning-phased clock genes. Plant Cell.

[CR165] Zagotta MT, Hicks KA, Jacobs CI, Young JC, Hangarter RP, Meeks-Wagner DR (1996). The Arabidopsis ELF3 gene regulates vegetative photomorphogenesis and the photoperiodic induction of flowering. Plant J.

[CR166] Zhang C, Gao M, Seitz NC, Angel W, Hallworth A, Wiratan L, Darwish O, Alkharouf N, Dawit T, Lin D, Egoshi R, Wang XP, McClung CR, Lu H (2019). LUX ARRHYTHMO mediates crosstalk between the circadian clock and defense in Arabidopsis. Nat Commun.

[CR167] Zhang C, Xie Q, Anderson RG, Ng G, Seitz NC, Peterson T, McClung CR, McDowell JM, Kong D, Kwak J, Lu H (2013). Crosstalk between the circadian clock and innate immunity in Arabidopsis. PLoS Pathog.

[CR168] Zhang LL, Li W, Tian YY, Davis SJ, Liu JX (2021). The E3 ligase XBAT35 mediates thermoresponsive hypocotyl growth by targeting ELF3 for degradation in Arabidopsis. J Integr Plant Biol.

[CR169] Zhang LL, Shao YJ, Ding L, Wang MJ, Davis SJ, Liu JX (2021). XBAT31 regulates thermoresponsive hypocotyl growth through mediating degradation of the thermosensor ELF3 in Arabidopsis. Sci Adv.

[CR170] Zhang S, Wu QR, Liu LL, Zhang HM, Gao JW, Pei ZM (2020). Osmotic stress alters circadian cytosolic ca(2+) oscillations and OSCA1 is required in circadian gated stress adaptation. Plant Signal Behav.

[CR171] Zhu JH, Lee BH, Dellinger M, Cui XP, Zhang CQ, Wu S, Nothnagel EA, Zhu JK (2010). A cellulose synthase-like protein is required for osmotic stress tolerance in Arabidopsis. Plant J.

[CR172] Zhu JK (2016). Abiotic stress signaling and responses in plants. Cell.

[CR173] Zhu JY, Oh E, Wang T, Wang ZY (2016). TOC1-PIF4 interaction mediates the circadian gating of thermoresponsive growth in Arabidopsis. Nat Commun.

[CR174] Zimmerman WF, Pittendrigh CS, Pavlidis T (1968). Temperature compensation of the circadian oscillation in *Drosophila pseudoobscura* and its entrainment by temperature cycles. J Insect Physiol.

